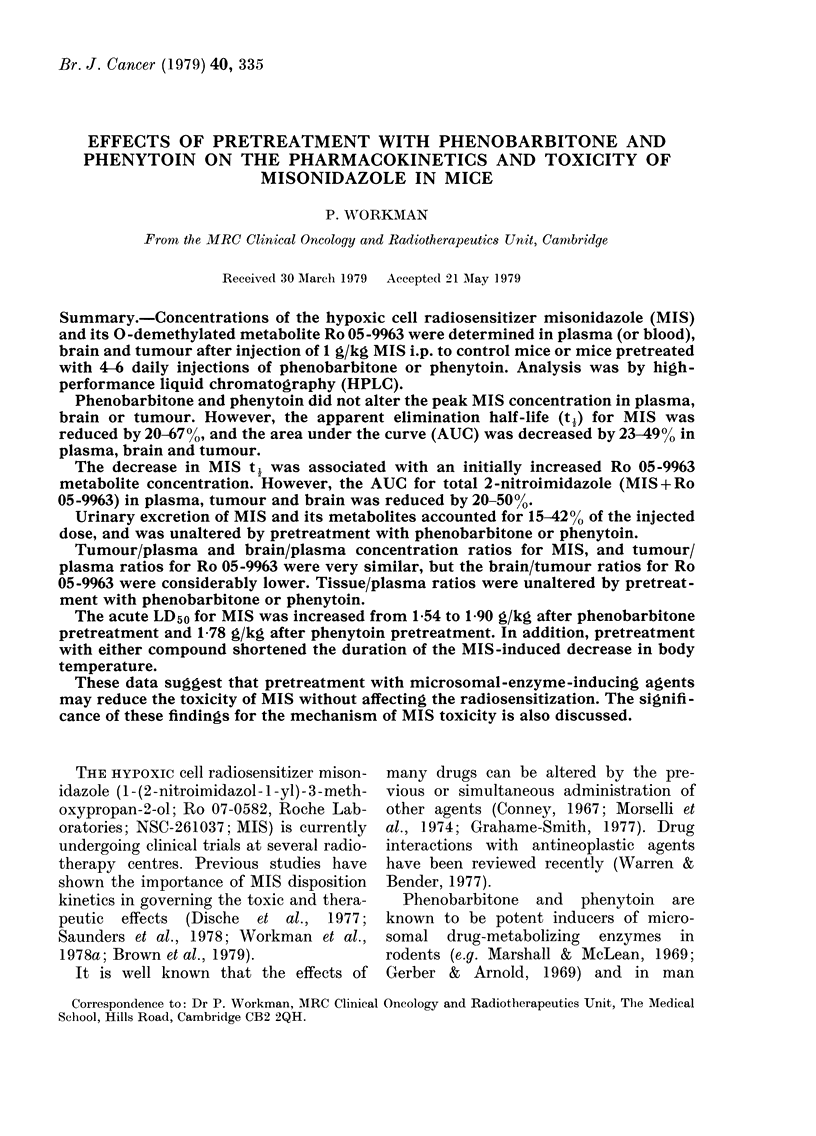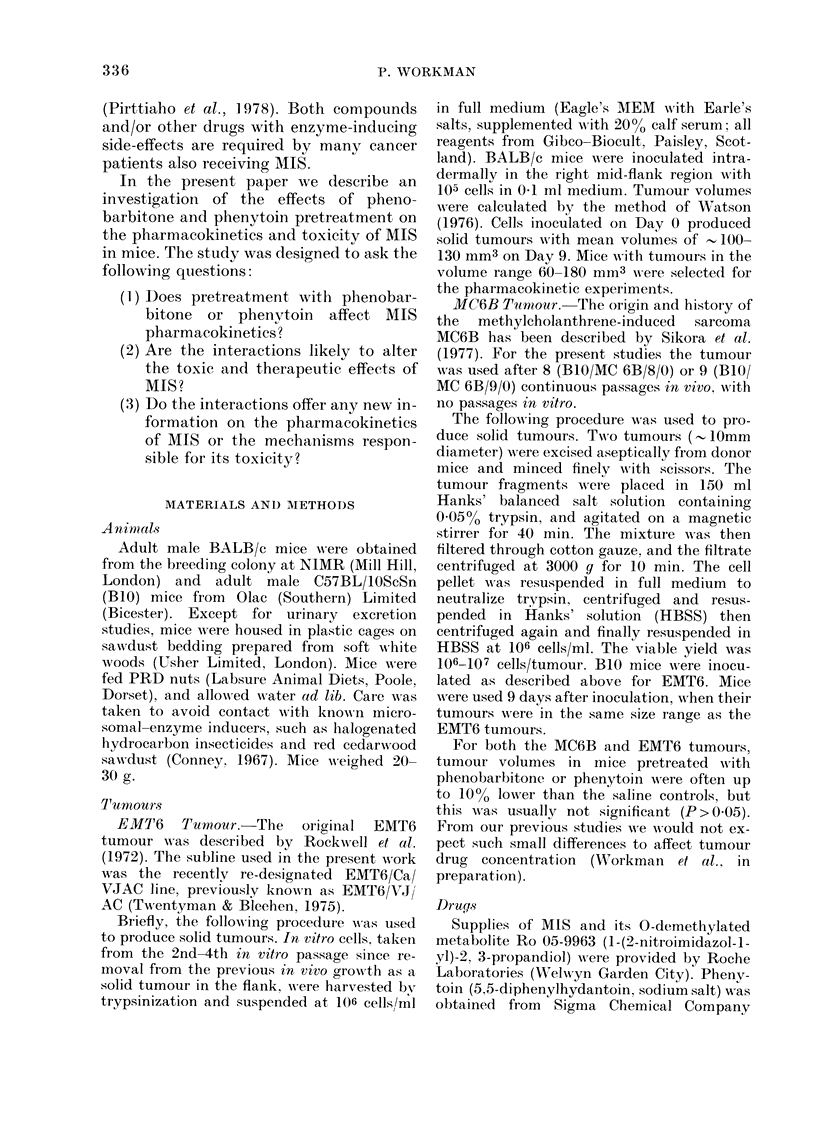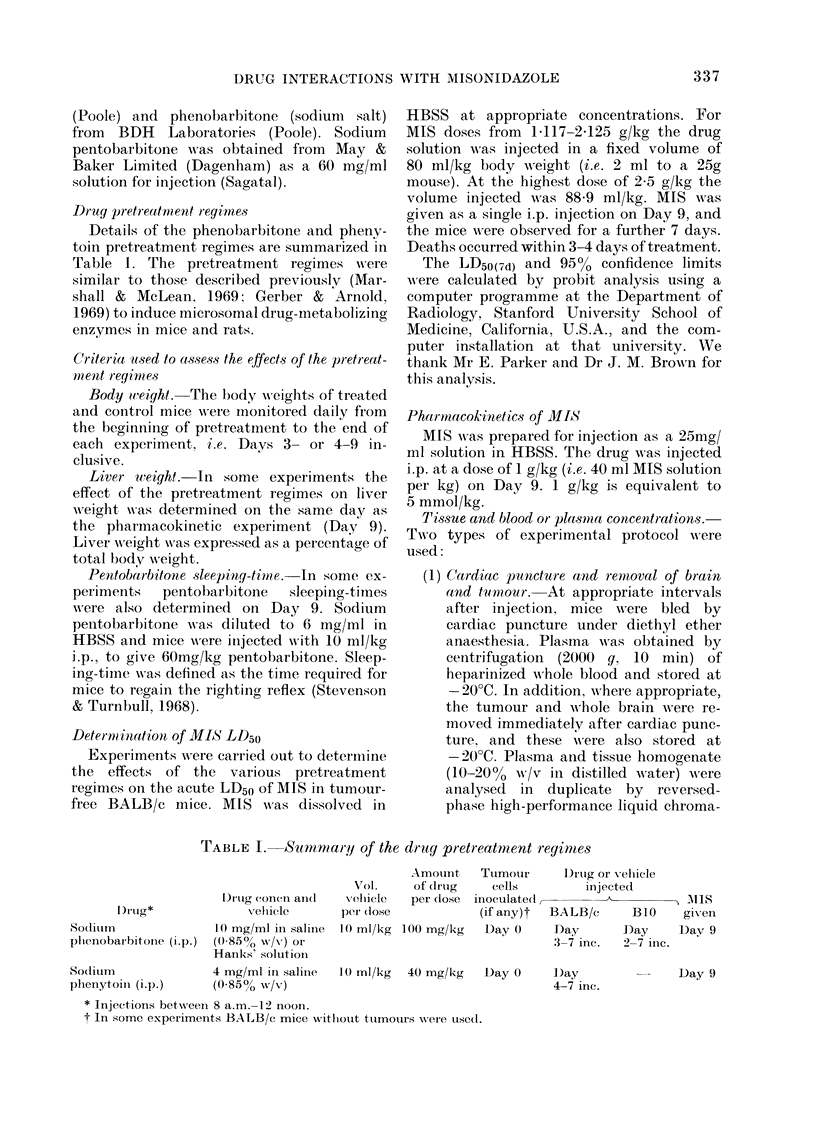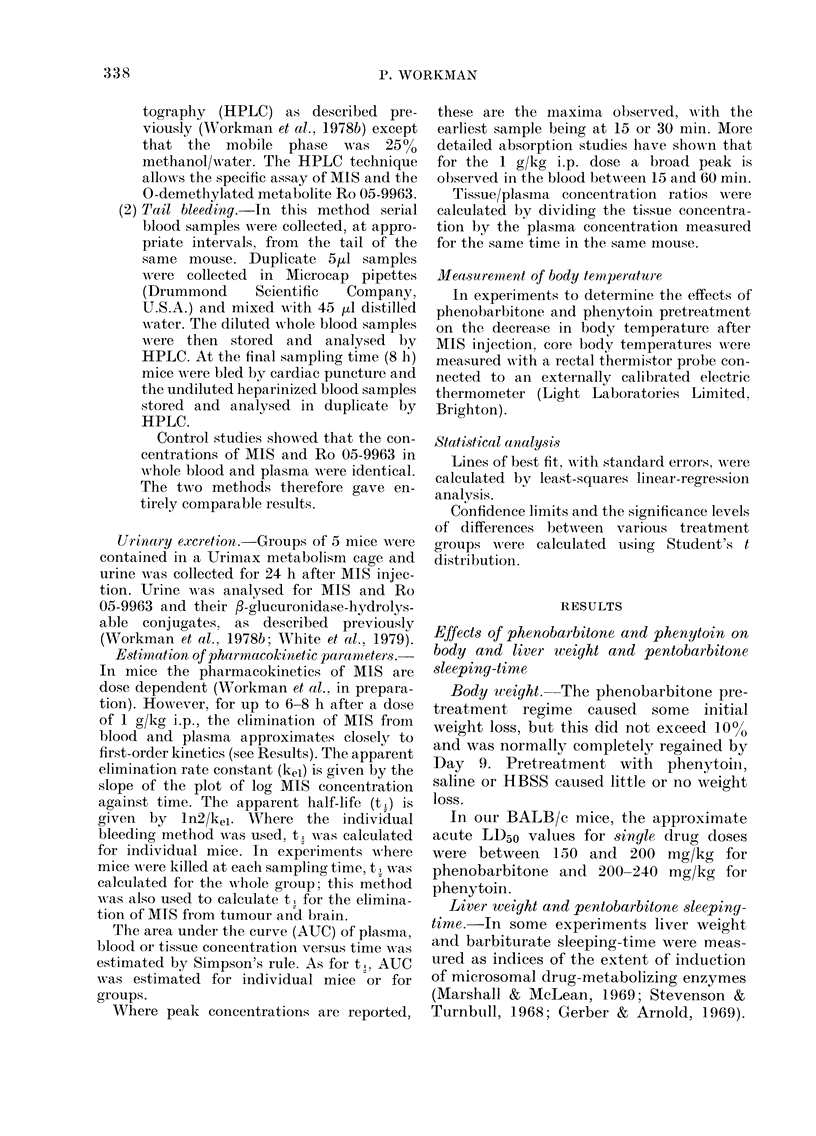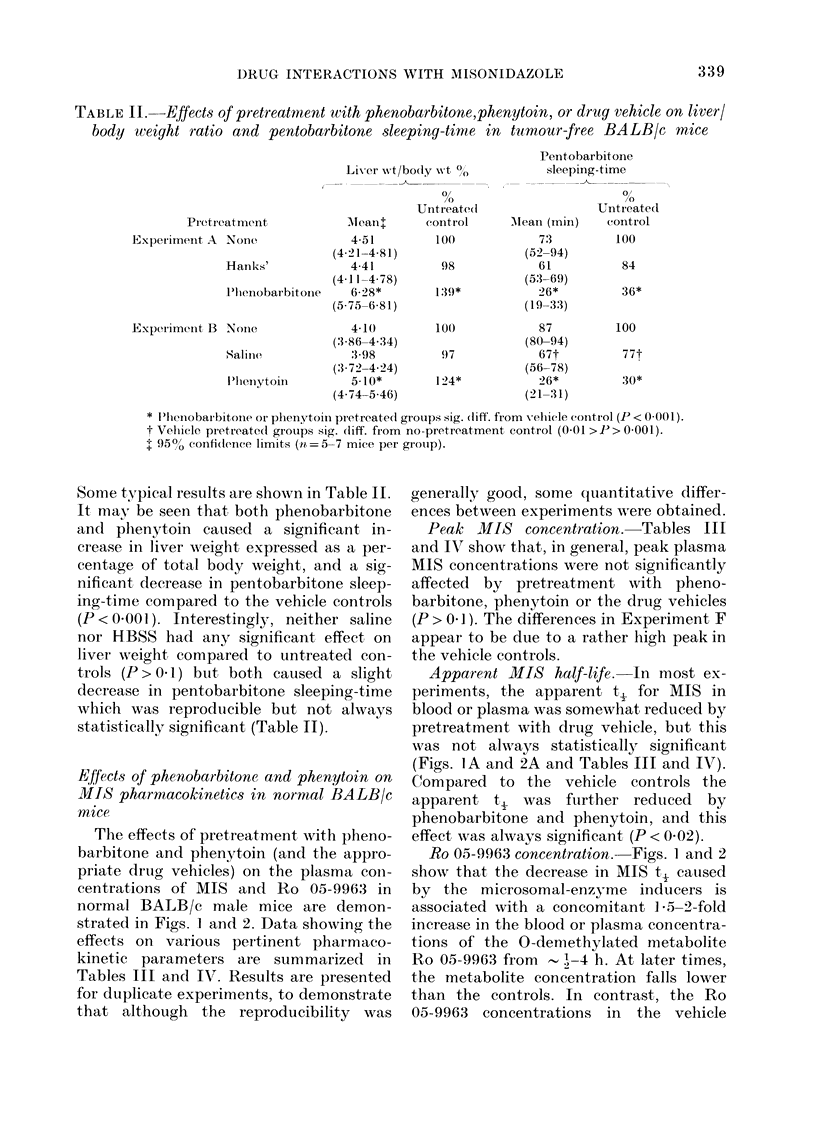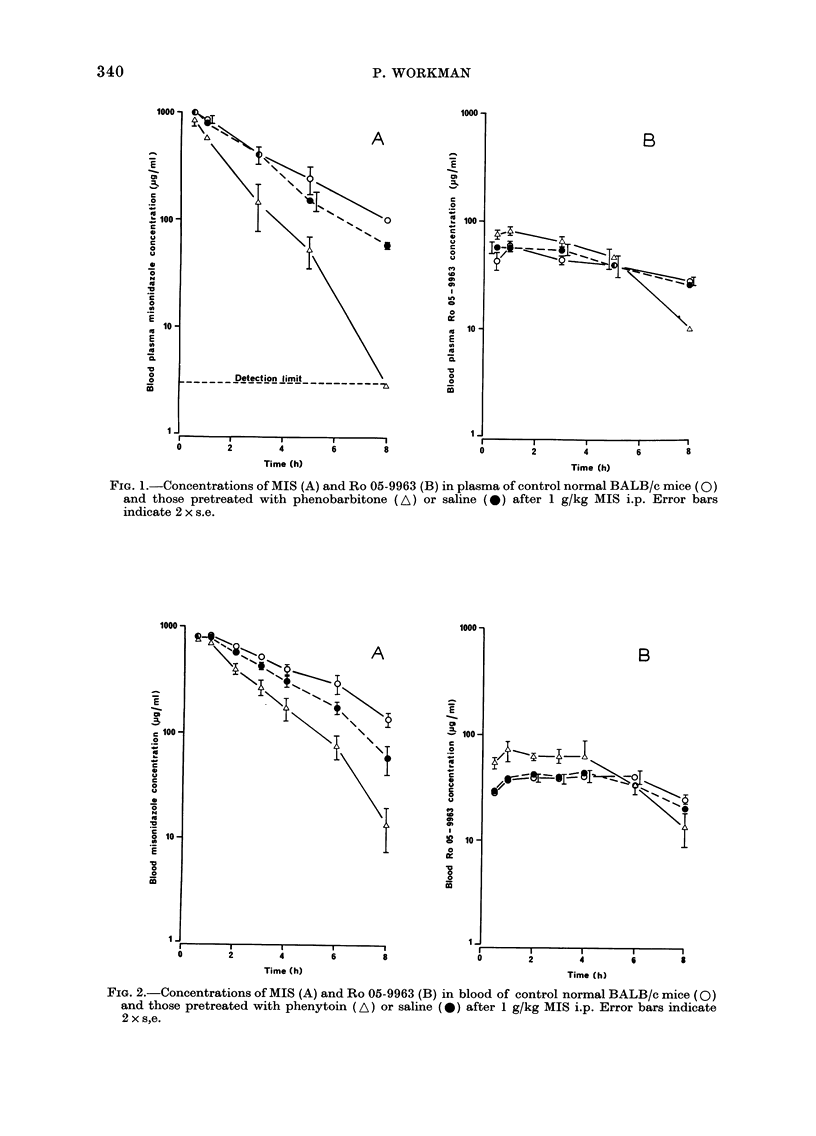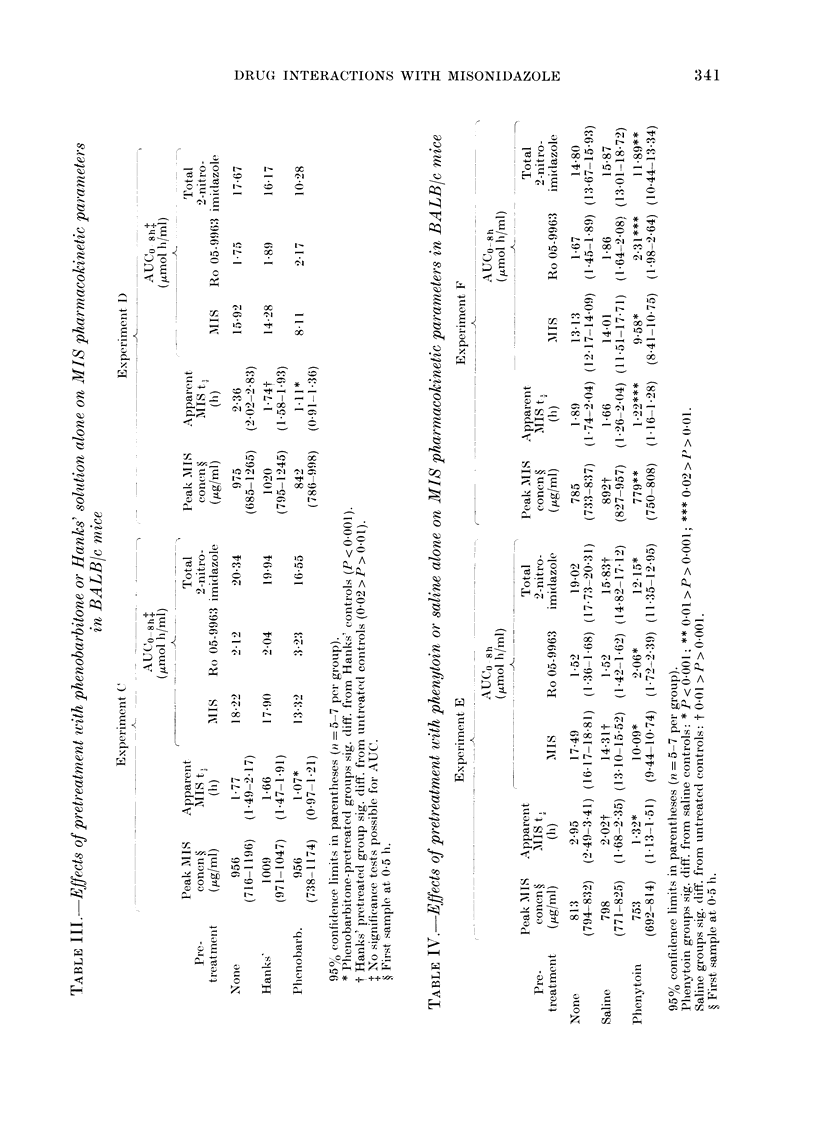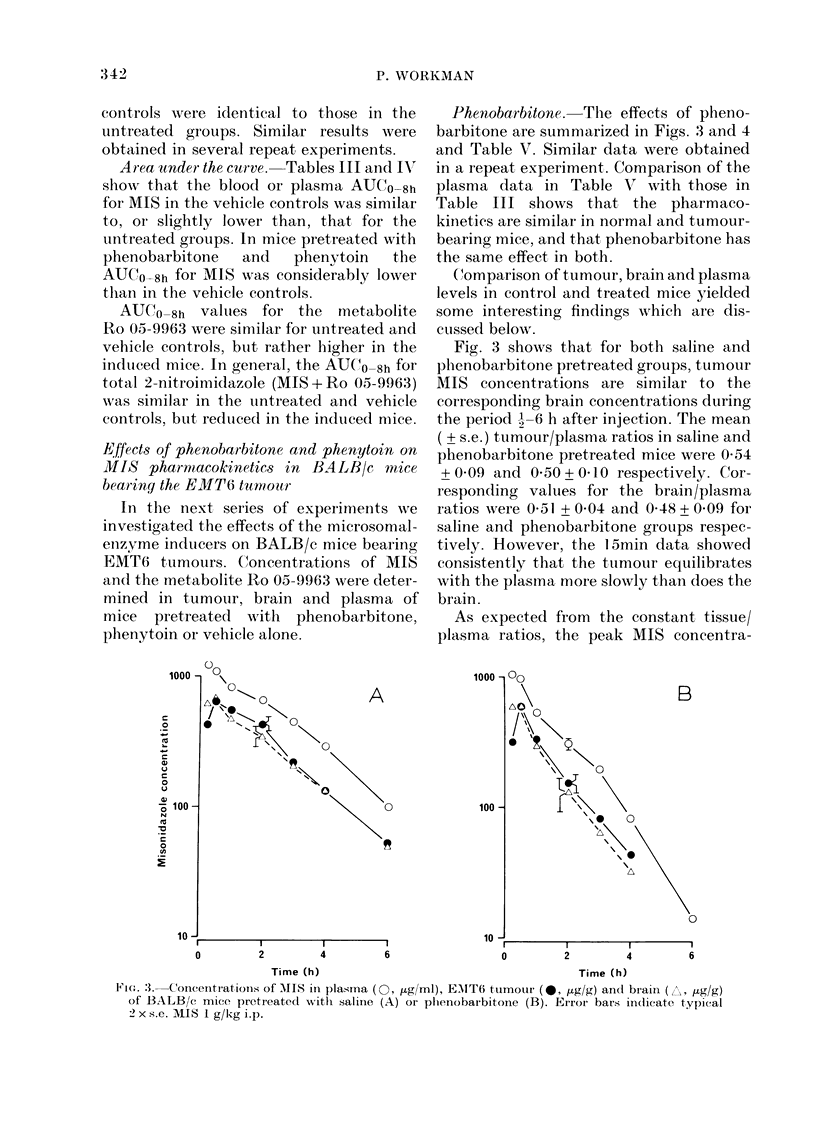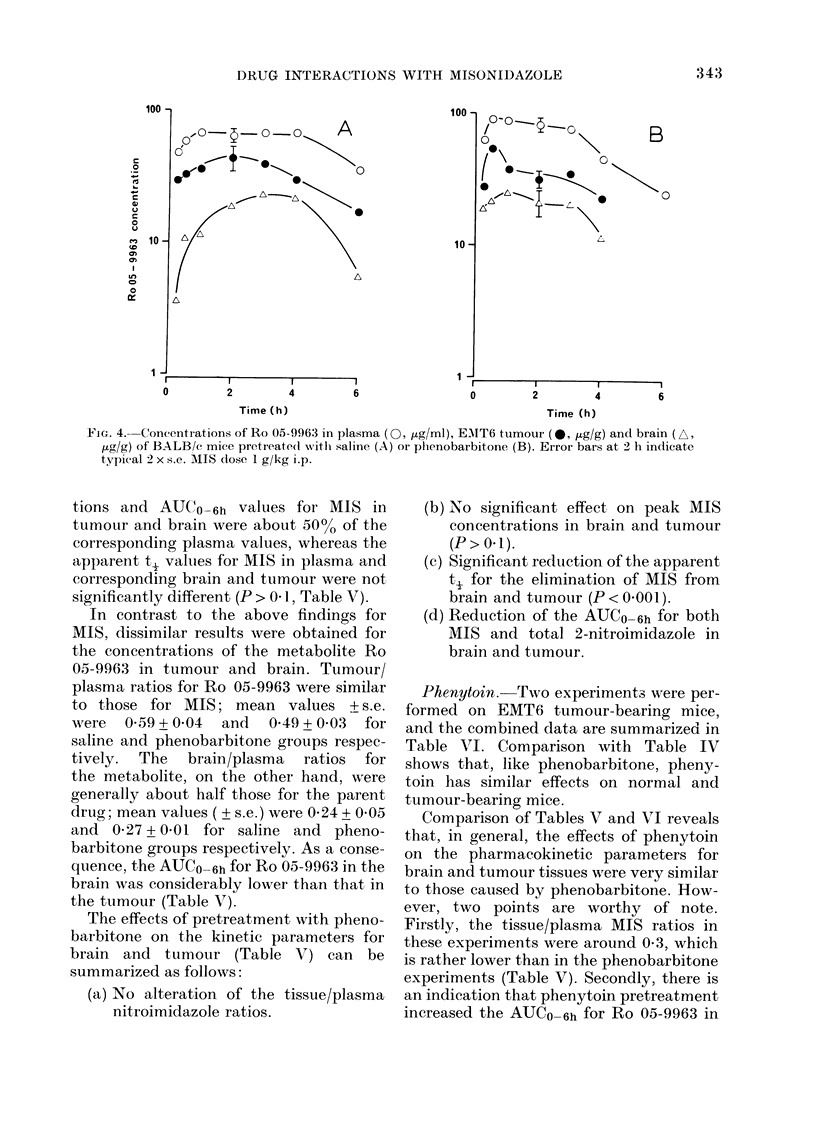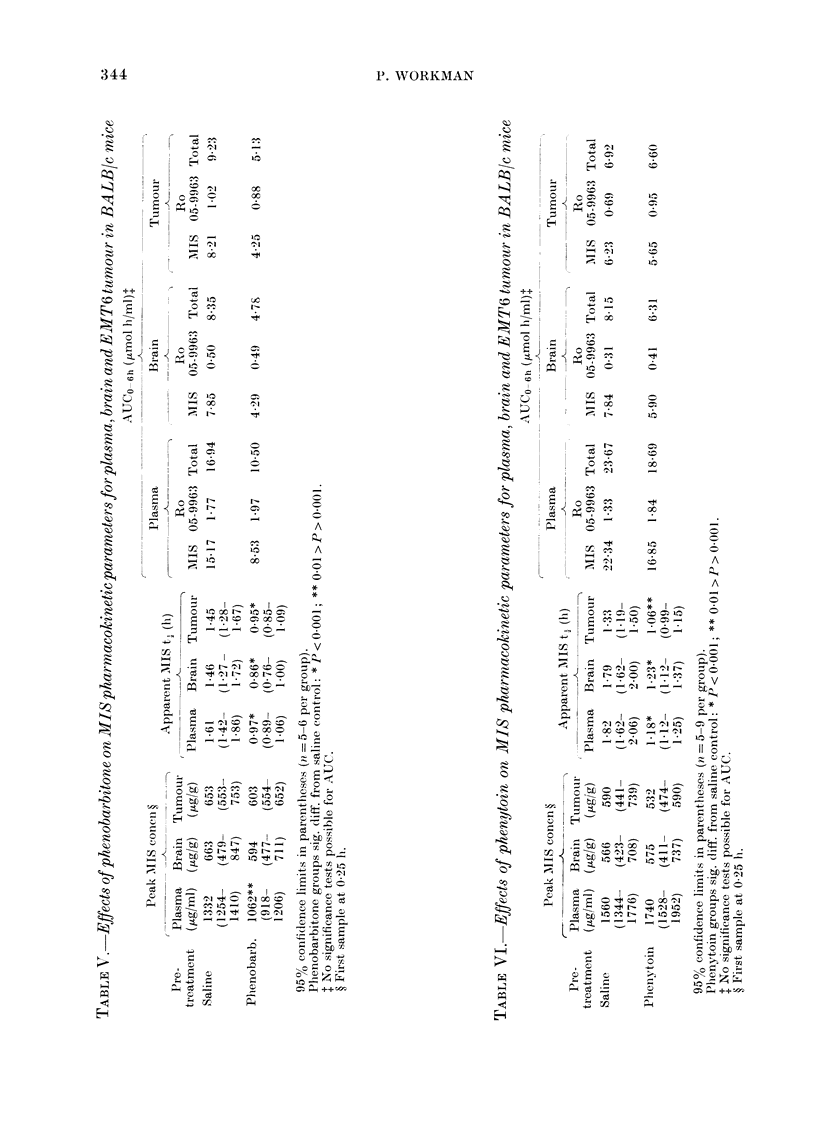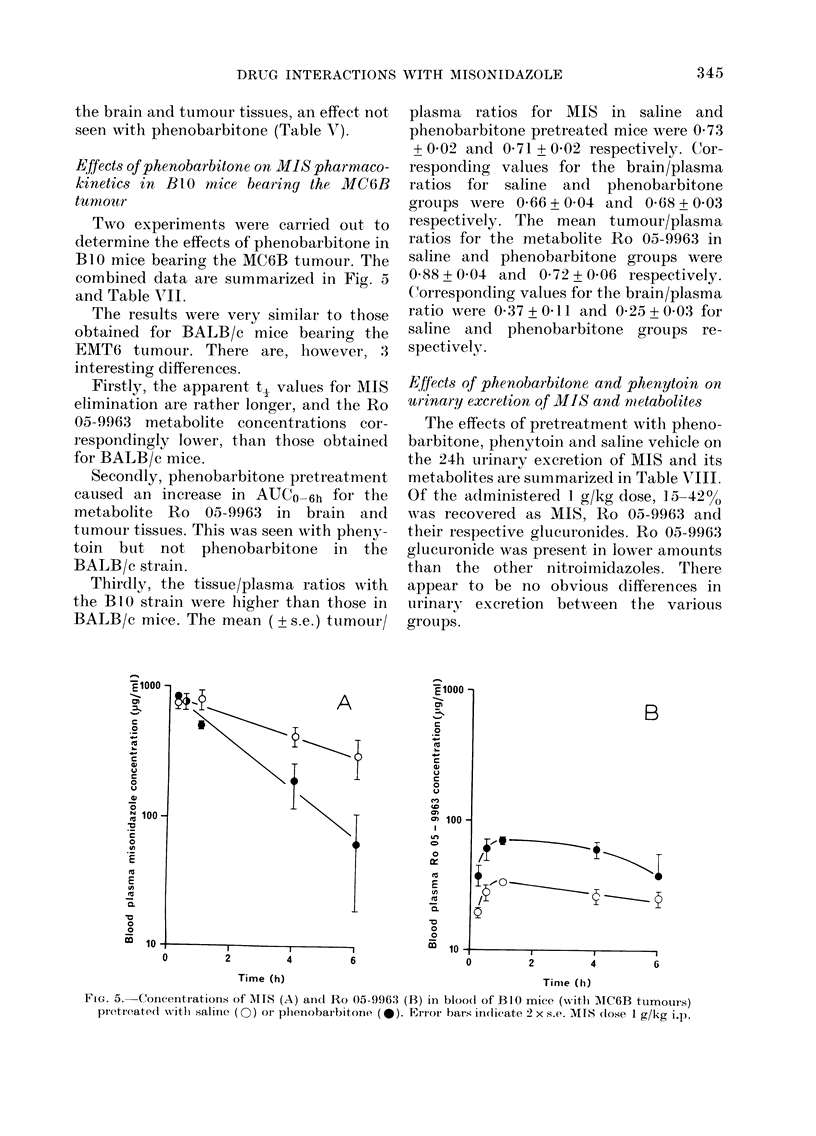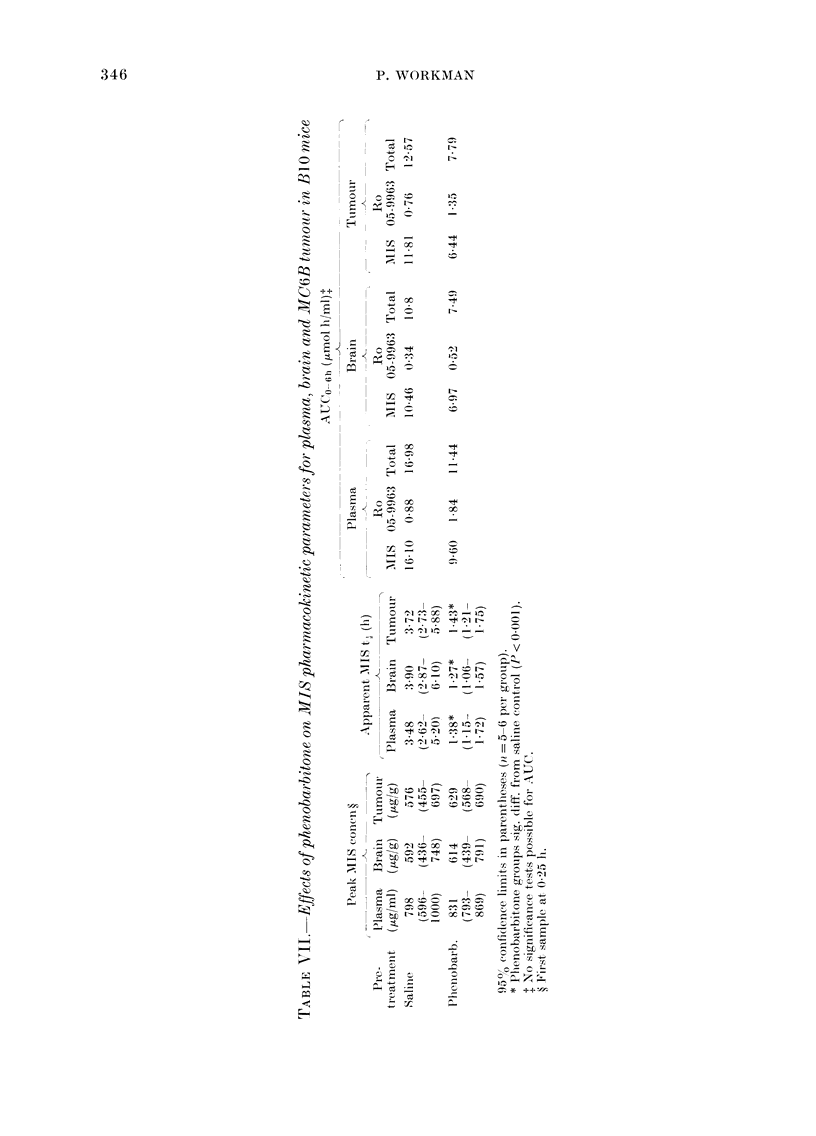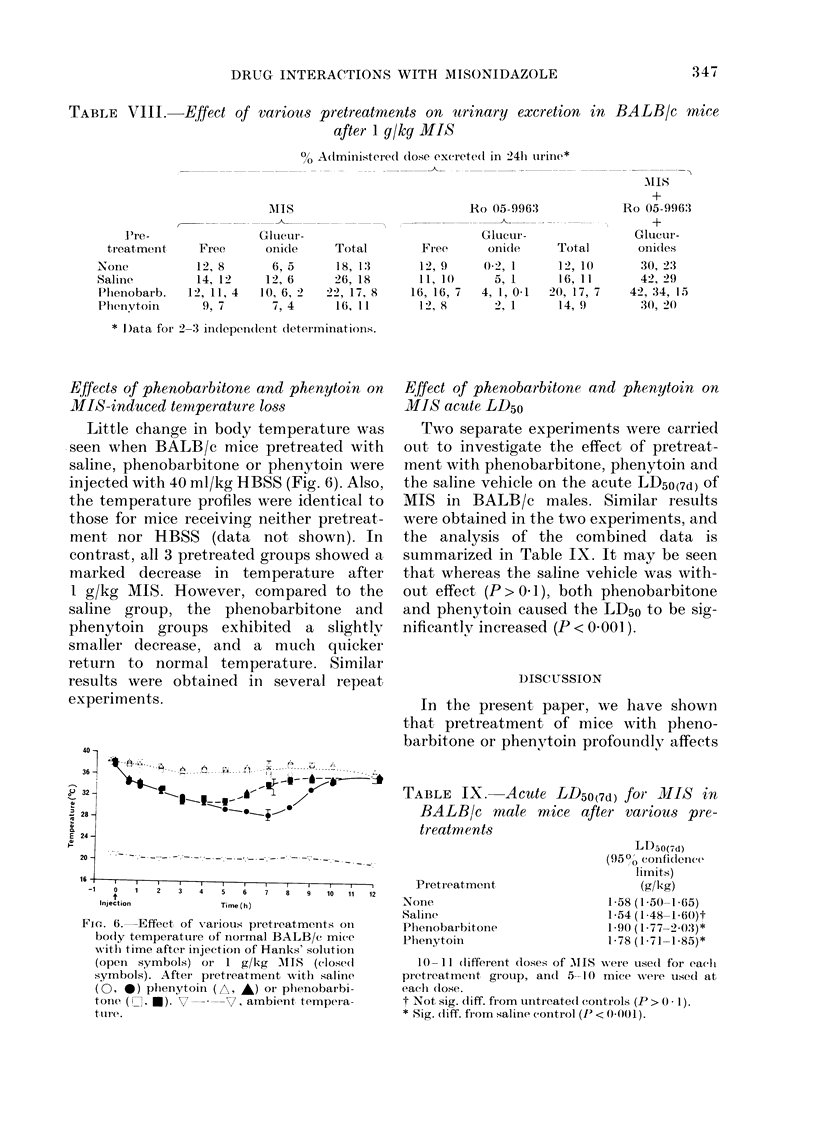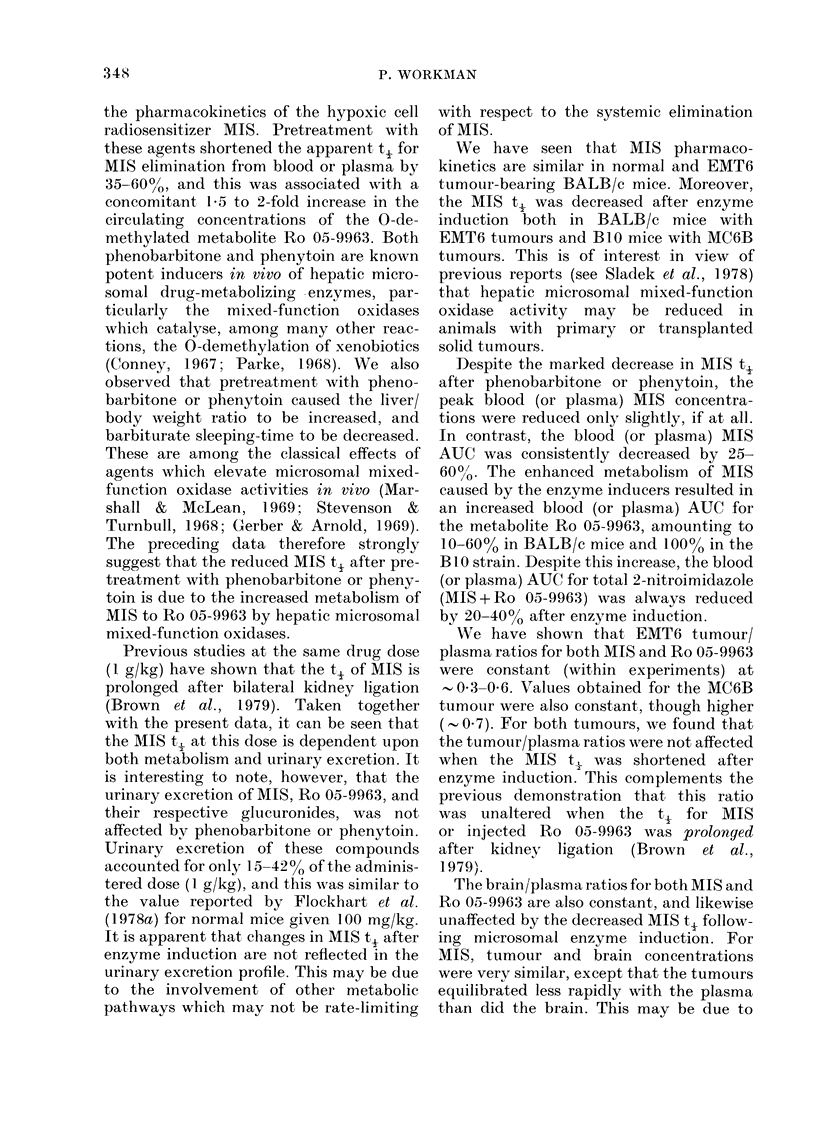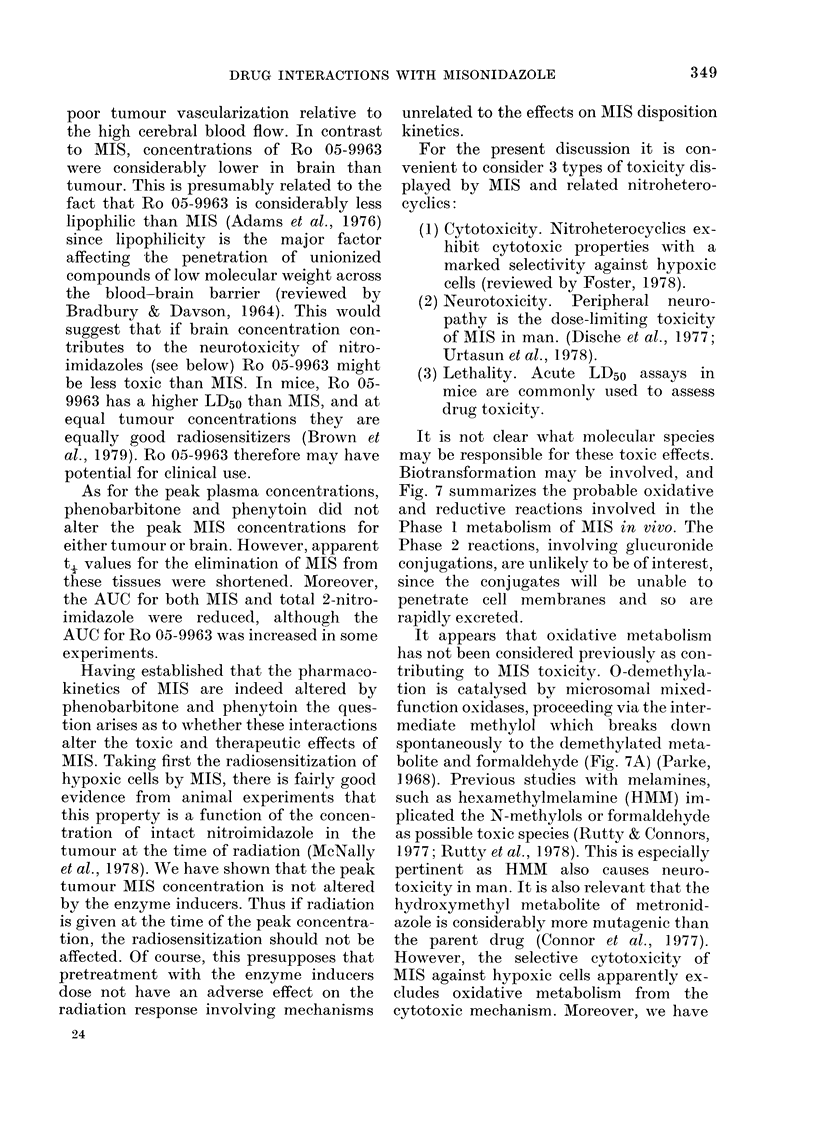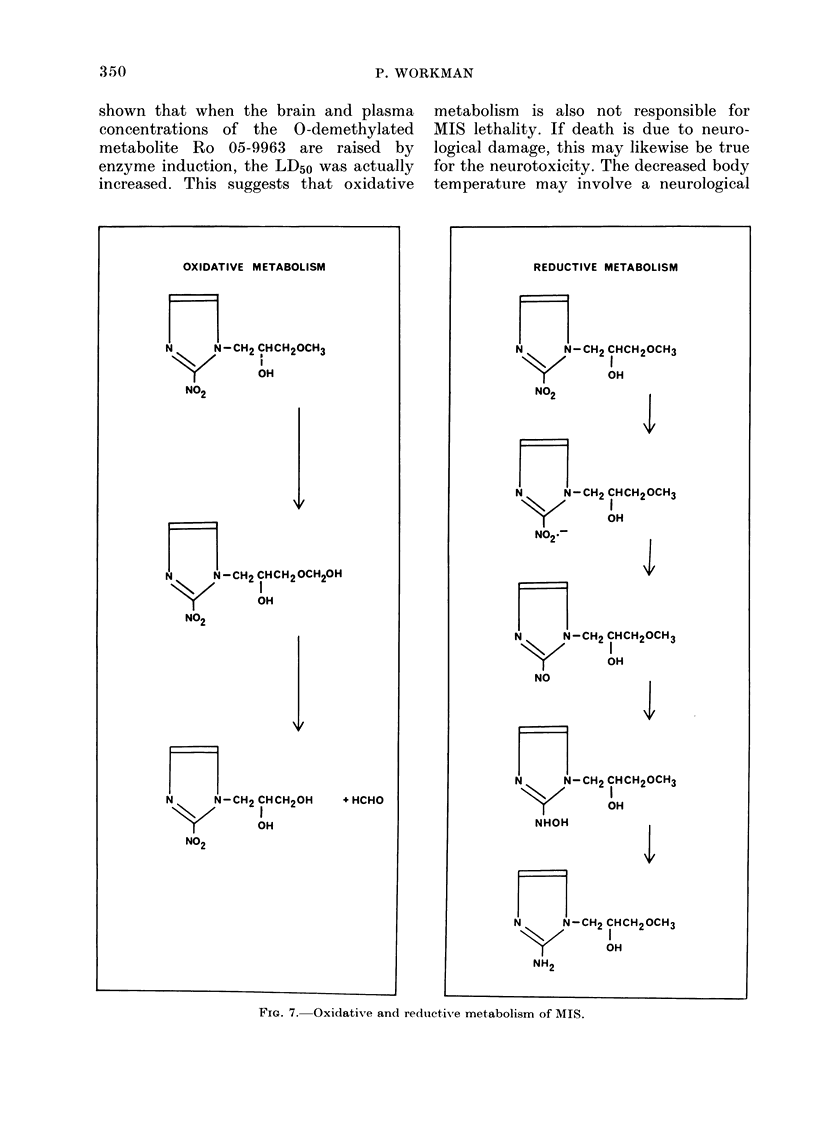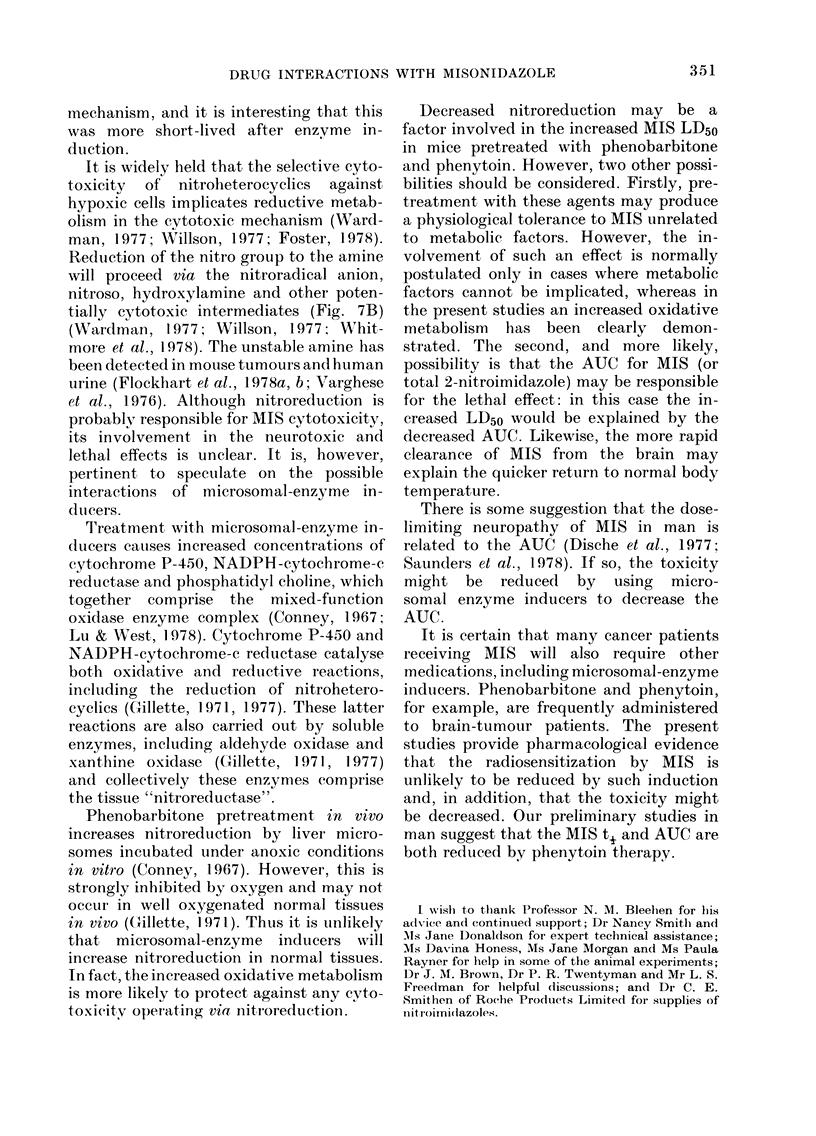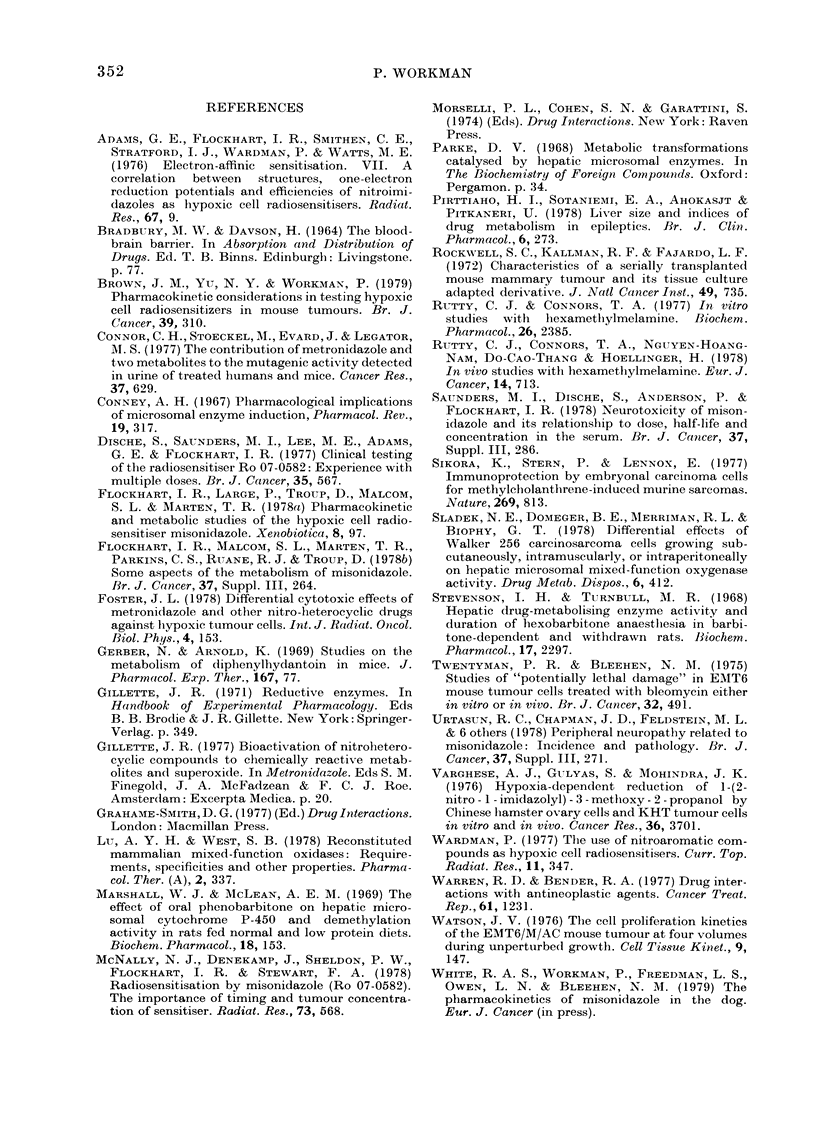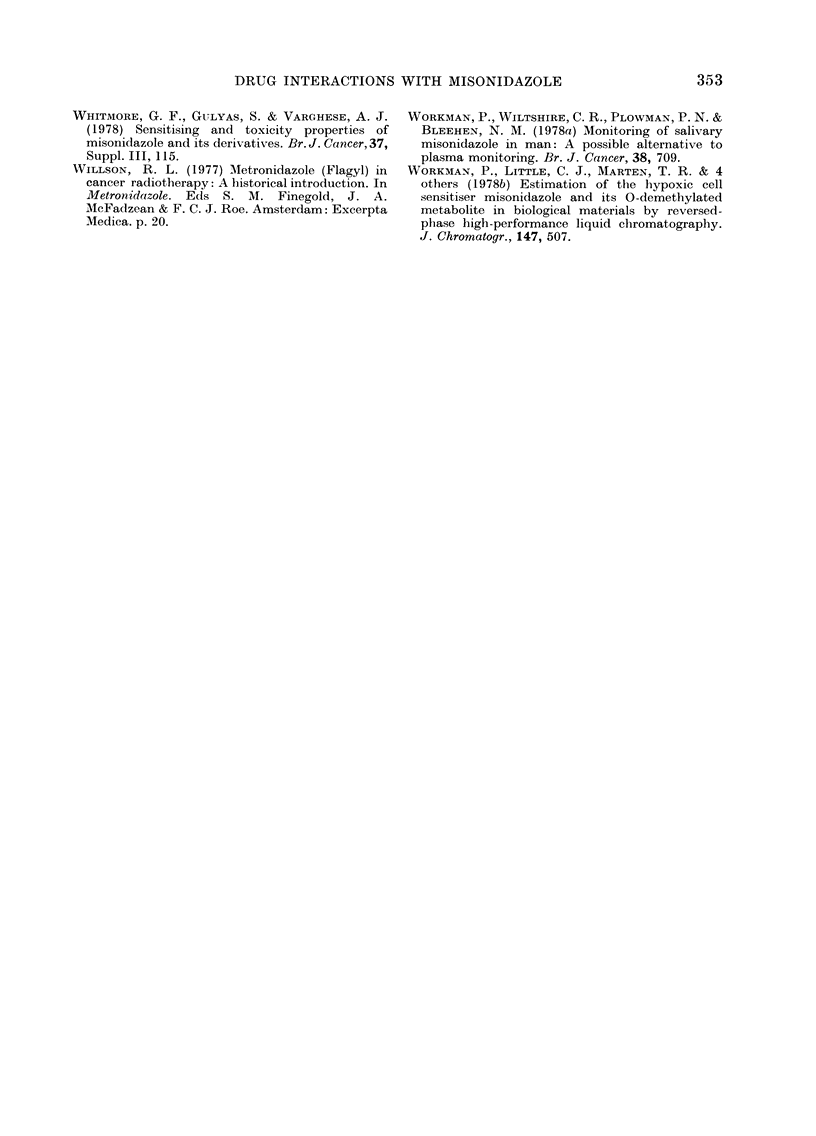# Effects of pretreatment with phenobarbitone and phenytoin on the pharmacokinetics and toxicity of phenytoin on the pharmacokinetics and toxicity of misonidazole in mice.

**DOI:** 10.1038/bjc.1979.187

**Published:** 1979-09

**Authors:** P. Workman

## Abstract

Concentrations of the hypoxic cell radiosensitizer misonidazole (MIS) and its O-demethylated metabolite Ro 05-9963 were determined in plasma (or blood), brain and tumour after injection of 1 g/kg MIS i.p. to control mice or mice pretreated with 4-6 daily injections of phenobarbitone or phenytoin. Analysis was by high-performance liquid chromatography (HPLC). Phenobarbitone and phenytoin did not alter the peak MIS concentration in plasma, brain or tumor. However, the apparent elimination half-life (t 1/2) for MIS was reduced by 20-67%, and the area under the curve (AUC) was decreased by 23-49% in plasma, brain and tumour. The decrease in MIS t 1/2 was associated with an initially increased Ro 05-9963 metabolite concentration. However, the AUC for total 2-nitromidazole (MIS + Ro 05-9963) in plasma, tumour and brain was reduced by 20-50%. Urinary excretion of MIS and its metabolites accounted for 15-42% of the injected dose, and was unaltered by pretreatment with phenobarbitone or phenytoin. Tumour/plasm and brain/plasma concentration ratios for MIS, and tumour/plasma ratios for Ro 05-9963 were very similar, but the brain/tumour ratios for Ro 05-9963 were considerably lower. Tissue/plasma ratios were unaltered by pretreatment with phenobarbitone or phenytoin. The acute LD50 for MIS was increased from 1.54 to 1.90 g/kg after phenobarbitone pretreatment and 1.78 g/kg after phenytoin pretreatment. In addition, pretreatment with either compound shortened the duration of the MIS-induced decrease in body temperature. These data suggest that pretreatment with microsomal-enzyme-inducing agents may reduce the toxicity of MIS without affecting the radiosensitization. The significance of these findings for the mechanism of MIS toxicity is also discussed.


*Br. J. Cancer* (1979) **40**, 335

### EFFECTS OF PRETREATMENT WITH PHENOBARBITONE AND PHENYTOIN ON THE PHARMACOKINETICS AND TOXICITY OF MISONIDAZOLE IN MICE

P. WORKMAN

*From the MRC Clinical Oncology and Radiotherapeutics Unit, Cambridge*

Received 30 March 1979 Accepted 21 May 1979

**Summary.—Concentrations of the hypoxic cell radiosensitizer misonidazole (MIS) and its O-demethylated metabolite Ro 05-9963 were determined in plasma (or blood), brain and tumour after injection of 1 g/kg MIS i.p. to control mice or mice pretreated with 4–6 daily injections of phenobarbitone or phenytoin. Analysis was by high-performance liquid chromatography (HPLC).**

**Phenobarbitone and phenytoin did not alter the peak MIS concentration in plasma, brain or tumour. However, the apparent elimination half-life ($t_{\frac{1}{2}}$) for MIS was reduced by 20–67%, and the area under the curve (AUC) was decreased by 23–49% in plasma, brain and tumour.**

**The decrease in MIS $t_{\frac{1}{2}}$ was associated with an initially increased Ro 05-9963 metabolite concentration. However, the AUC for total 2-nitroimidazole (MIS+Ro 05-9963) in plasma, tumour and brain was reduced by 20–50%.**

**Urinary excretion of MIS and its metabolites accounted for 15–42% of the injected dose, and was unaltered by pretreatment with phenobarbitone or phenytoin.**

**Tumour/plasma and brain/plasma concentration ratios for MIS, and tumour/plasma ratios for Ro 05-9963 were very similar, but the brain/tumour ratios for Ro 05-9963 were considerably lower. Tissue/plasma ratios were unaltered by pretreatment with phenobarbitone or phenytoin.**

**The acute $LD_{50}$ for MIS was increased from 1·54 to 1·90 g/kg after phenobarbitone pretreatment and 1·78 g/kg after phenytoin pretreatment. In addition, pretreatment with either compound shortened the duration of the MIS-induced decrease in body temperature.**

**These data suggest that pretreatment with microsomal-enzyme-inducing agents may reduce the toxicity of MIS without affecting the radiosensitization. The significance of these findings for the mechanism of MIS toxicity is also discussed.**

THE HYPOXIC cell radiosensitizer misonidazole (1-(2-nitroimidazol-1-yl)-3-methoxypropan-2-ol; Ro 07-0582, Roche Laboratories; NSC-261037; MIS) is currently undergoing clinical trials at several radiotherapy centres. Previous studies have shown the importance of MIS disposition kinetics in governing the toxic and therapeutic effects (Dische *et al.*, 1977; Saunders *et al.*, 1978; Workman *et al.*, 1978*a*; Brown *et al.*, 1979).

It is well known that the effects of many drugs can be altered by the previous or simultaneous administration of other agents (Conney, 1967; Morselli *et al.*, 1974; Grahame-Smith, 1977). Drug interactions with antineoplastic agents have been reviewed recently (Warren & Bender, 1977).

Phenobarbitone and phenytoin are known to be potent inducers of microsomal drug-metabolizing enzymes in rodents (*e.g.* Marshall & McLean, 1969; Gerber & Arnold, 1969) and in man

Correspondence to: Dr P. Workman, MRC Clinical Oncology and Radiotherapeutics Unit, The Medical School, Hills Road, Cambridge CB2 2QH.

(Pirttiaho *et al.*, 1978). Both compounds and/or other drugs with enzyme-inducing side-effects are required by many cancer patients also receiving MIS.

In the present paper we describe an investigation of the effects of phenobarbitone and phenytoin pretreatment on the pharmacokinetics and toxicity of MIS in mice. The study was designed to ask the following questions:

(1) Does pretreatment with phenobarbitone or phenytoin affect MIS pharmacokinetics?
(2) Are the interactions likely to alter the toxic and therapeutic effects of MIS?
(3) Do the interactions offer any new information on the pharmacokinetics of MIS or the mechanisms responsible for its toxicity?

### MATERIALS AND METHODS

### *Animals*

Adult male BALB/c mice were obtained from the breeding colony at NIMR (Mill Hill, London) and adult male C57BL/10ScSn (B10) mice from Olac (Southern) Limited (Bicester). Except for urinary excretion studies, mice were housed in plastic cages on sawdust bedding prepared from soft white woods (Usher Limited, London). Mice were fed PRD nuts (Labsure Animal Diets, Poole, Dorset), and allowed water *ad lib.* Care was taken to avoid contact with known microsomal-enzyme inducers, such as halogenated hydrocarbon insecticides and red cedarwood sawdust (Conney, 1967). Mice weighed 20–30 g.

### *Tumours*

*EMT*6 *Tumour*.—The original EMT6 tumour was described by Rockwell *et al.* (1972). The subline used in the present work was the recently re-designated EMT6/Ca/VJAC line, previously known as EMT6/VJ/AC (Twentyman & Bleehen, 1975).

Briefly, the following procedure was used to produce solid tumours. *In vitro* cells, taken from the 2nd–4th *in vitro* passage since removal from the previous *in vivo* growth as a solid tumour in the flank, were harvested by trypsinization and suspended at $10^6$ cells/ml in full medium (Eagle's MEM with Earle's salts, supplemented with 20% calf serum; all reagents from Gibco–Biocult, Paisley, Scotland). BALB/c mice were inoculated intradermally in the right mid-flank region with $10^5$ cells in 0·1 ml medium. Tumour volumes were calculated by the method of Watson (1976). Cells inoculated on Day 0 produced solid tumours with mean volumes of ~100–130 mm³ on Day 9. Mice with tumours in the volume range 60–180 mm³ were selected for the pharmacokinetic experiments.

*MC6B Tumour*.—The origin and history of the methylcholanthrene-induced sarcoma MC6B has been described by Sikora *et al.* (1977). For the present studies the tumour was used after 8 (B10/MC 6B/8/0) or 9 (B10/MC 6B/9/0) continuous passages *in vivo*, with no passages *in vitro*.

The following procedure was used to produce solid tumours. Two tumours (~10mm diameter) were excised aseptically from donor mice and minced finely with scissors. The tumour fragments were placed in 150 ml Hanks' balanced salt solution containing 0·05% trypsin, and agitated on a magnetic stirrer for 40 min. The mixture was then filtered through cotton gauze, and the filtrate centrifuged at 3000 *g* for 10 min. The cell pellet was resuspended in full medium to neutralize trypsin, centrifuged and resuspended in Hanks' solution (HBSS) then centrifuged again and finally resuspended in HBSS at $10^6$ cells/ml. The viable yield was $10^6$–$10^7$ cells/tumour. B10 mice were inoculated as described above for EMT6. Mice were used 9 days after inoculation, when their tumours were in the same size range as the EMT6 tumours.

For both the MC6B and EMT6 tumours, tumour volumes in mice pretreated with phenobarbitone or phenytoin were often up to 10% lower than the saline controls, but this was usually not significant ($P > 0{\cdot}05$). From our previous studies we would not expect such small differences to affect tumour drug concentration (Workman *et al.*, in preparation).

### *Drugs*

Supplies of MIS and its O-demethylated metabolite Ro 05-9963 (1-(2-nitroimidazol-1-yl)-2, 3-propandiol) were provided by Roche Laboratories (Welwyn Garden City). Phenytoin (5,5-diphenylhydantoin, sodium salt) was obtained from Sigma Chemical Company

(Poole) and phenobarbitone (sodium salt) from BDH Laboratories (Poole). Sodium pentobarbitone was obtained from May & Baker Limited (Dagenham) as a 60 mg/ml solution for injection (Sagatal).

*Drug pretreatment regimes*

Details of the phenobarbitone and phenytoin pretreatment regimes are summarized in Table I. The pretreatment regimes were similar to those described previously (Marshall & McLean, 1969; Gerber & Arnold, 1969) to induce microsomal drug-metabolizing enzymes in mice and rats.

*Criteria used to assess the effects of the pretreatment regimes*

*Body weight*.—The body weights of treated and control mice were monitored daily from the beginning of pretreatment to the end of each experiment, *i.e.* Days 3- or 4–9 inclusive.

*Liver weight*.—In some experiments the effect of the pretreatment regimes on liver weight was determined on the same day as the pharmacokinetic experiment (Day 9). Liver weight was expressed as a percentage of total body weight.

*Pentobarbitone sleeping-time*.—In some experiments pentobarbitone sleeping-times were also determined on Day 9. Sodium pentobarbitone was diluted to 6 mg/ml in HBSS and mice were injected with 10 ml/kg i.p., to give 60mg/kg pentobarbitone. Sleeping-time was defined as the time required for mice to regain the righting reflex (Stevenson & Turnbull, 1968).

*Determination of MIS $LD_{50}$*

Experiments were carried out to determine the effects of the various pretreatment regimes on the acute $LD_{50}$ of MIS in tumour-free BALB/c mice. MIS was dissolved in HBSS at appropriate concentrations. For MIS doses from 1·117–2·125 g/kg the drug solution was injected in a fixed volume of 80 ml/kg body weight (*i.e.* 2 ml to a 25g mouse). At the highest dose of 2·5 g/kg the volume injected was 88·9 ml/kg. MIS was given as a single i.p. injection on Day 9, and the mice were observed for a further 7 days. Deaths occurred within 3–4 days of treatment.

The $LD_{50(7d)}$ and 95% confidence limits were calculated by probit analysis using a computer programme at the Department of Radiology, Stanford University School of Medicine, California, U.S.A., and the computer installation at that university. We thank Mr E. Parker and Dr J. M. Brown for this analysis.

*Pharmacokinetics of MIS*

MIS was prepared for injection as a 25mg/ml solution in HBSS. The drug was injected i.p. at a dose of 1 g/kg (*i.e.* 40 ml MIS solution per kg) on Day 9. 1 g/kg is equivalent to 5 mmol/kg.

*Tissue and blood or plasma concentrations*.—Two types of experimental protocol were used:

(1) *Cardiac puncture and removal of brain and tumour*.—At appropriate intervals after injection, mice were bled by cardiac puncture under diethyl ether anaesthesia. Plasma was obtained by centrifugation (2000 *g*, 10 min) of heparinized whole blood and stored at −20°C. In addition, where appropriate, the tumour and whole brain were removed immediately after cardiac puncture, and these were also stored at −20°C. Plasma and tissue homogenate (10–20% w/v in distilled water) were analysed in duplicate by reversed-phase high-performance liquid chroma-

TABLE I.—*Summary of the drug pretreatment regimes*

| Drug* | Drug concn and vehicle | Vol. vehicle per dose | Amount of drug per dose | Tumour cells inoculated (if any)† | Drug or vehicle injected: BALB/c | Drug or vehicle injected: B10 | MIS given |
|---|---|---|---|---|---|---|---|
| Sodium phenobarbitone (i.p.) | 10 mg/ml in saline (0·85% w/v) or Hanks' solution | 10 ml/kg | 100 mg/kg | Day 0 | Day 3–7 inc. | Day 2–7 inc. | Day 9 |
| Sodium phenytoin (i.p.) | 4 mg/ml in saline (0·85% w/v) | 10 ml/kg | 40 mg/kg | Day 0 | Day 4–7 inc. | — | Day 9 |

* Injections between 8 a.m.–12 noon.

† In some experiments BALB/c mice without tumours were used.

tography (HPLC) as described previously (Workman *et al.*, 1978*b*) except that the mobile phase was 25% methanol/water. The HPLC technique allows the specific assay of MIS and the O-demethylated metabolite Ro 05-9963.

(2) *Tail bleeding.*—In this method serial blood samples were collected, at appropriate intervals, from the tail of the same mouse. Duplicate 5$\mu$l samples were collected in Microcap pipettes (Drummond Scientific Company, U.S.A.) and mixed with 45 $\mu$l distilled water. The diluted whole blood samples were then stored and analysed by HPLC. At the final sampling time (8 h) mice were bled by cardiac puncture and the undiluted heparinized blood samples stored and analysed in duplicate by HPLC.

Control studies showed that the concentrations of MIS and Ro 05-9963 in whole blood and plasma were identical. The two methods therefore gave entirely comparable results.

*Urinary excretion.*—Groups of 5 mice were contained in a Urimax metabolism cage and urine was collected for 24 h after MIS injection. Urine was analysed for MIS and Ro 05-9963 and their $\beta$-glucuronidase-hydrolysable conjugates, as described previously (Workman *et al.*, 1978*b*; White *et al.*, 1979).

*Estimation of pharmacokinetic parameters.*—In mice the pharmacokinetics of MIS are dose dependent (Workman *et al.*, in preparation). However, for up to 6–8 h after a dose of 1 g/kg i.p., the elimination of MIS from blood and plasma approximates closely to first-order kinetics (see Results). The apparent elimination rate constant ($k_{el}$) is given by the slope of the plot of log MIS concentration against time. The apparent half-life ($t_{\frac{1}{2}}$) is given by $\ln 2/k_{el}$. Where the individual bleeding method was used, $t_{\frac{1}{2}}$ was calculated for individual mice. In experiments where mice were killed at each sampling time, $t_{\frac{1}{2}}$ was calculated for the whole group; this method was also used to calculate $t_{\frac{1}{2}}$ for the elimination of MIS from tumour and brain.

The area under the curve (AUC) of plasma, blood or tissue concentration versus time was estimated by Simpson's rule. As for $t_{\frac{1}{2}}$, AUC was estimated for individual mice or for groups.

Where peak concentrations are reported, these are the maxima observed, with the earliest sample being at 15 or 30 min. More detailed absorption studies have shown that for the 1 g/kg i.p. dose a broad peak is observed in the blood between 15 and 60 min.

Tissue/plasma concentration ratios were calculated by dividing the tissue concentration by the plasma concentration measured for the same time in the same mouse.

*Measurement of body temperature*

In experiments to determine the effects of phenobarbitone and phenytoin pretreatment on the decrease in body temperature after MIS injection, core body temperatures were measured with a rectal thermistor probe connected to an externally calibrated electric thermometer (Light Laboratories Limited, Brighton).

*Statistical analysis*

Lines of best fit, with standard errors, were calculated by least-squares linear-regression analysis.

Confidence limits and the significance levels of differences between various treatment groups were calculated using Student's *t* distribution.

### RESULTS

*Effects of phenobarbitone and phenytoin on body and liver weight and pentobarbitone sleeping-time*

*Body weight.*—The phenobarbitone pretreatment regime caused some initial weight loss, but this did not exceed 10% and was normally completely regained by Day 9. Pretreatment with phenytoin, saline or HBSS caused little or no weight loss.

In our BALB/c mice, the approximate acute $LD_{50}$ values for *single* drug doses were between 150 and 200 mg/kg for phenobarbitone and 200–240 mg/kg for phenytoin.

*Liver weight and pentobarbitone sleeping-time.*—In some experiments liver weight and barbiturate sleeping-time were measured as indices of the extent of induction of microsomal drug-metabolizing enzymes (Marshall & McLean, 1969; Stevenson & Turnbull, 1968; Gerber & Arnold, 1969).

TABLE II.—*Effects of pretreatment with phenobarbitone, phenytoin, or drug vehicle on liver/body weight ratio and pentobarbitone sleeping-time in tumour-free BALB/c mice*

| | | Liver wt/body wt % | | Pentobarbitone sleeping-time | |
|---|---|---|---|---|---|
| Pretreatment | | Mean‡ | % Untreated control | Mean (min) | % Untreated control |
| Experiment A | None | 4·51 (4·21–4·81) | 100 | 73 (52–94) | 100 |
| | Hanks' | 4·41 (4·11–4·78) | 98 | 61 (53–69) | 84 |
| | Phenobarbitone | 6·28* (5·75–6·81) | 139* | 26* (19–33) | 36* |
| Experiment B | None | 4·10 (3·86–4·34) | 100 | 87 (80–94) | 100 |
| | Saline | 3·98 (3·72–4·24) | 97 | 67† (56–78) | 77† |
| | Phenytoin | 5·10* (4·74–5·46) | 124* | 26* (21–31) | 30* |

* Phenobarbitone or phenytoin pretreated groups sig. diff. from vehicle control ($P < 0{\cdot}001$).

† Vehicle pretreated groups sig. diff. from no-pretreatment control ($0{\cdot}01 > P > 0{\cdot}001$).

‡ 95% confidence limits ($n = 5$–7 mice per group).

Some typical results are shown in Table II. It may be seen that both phenobarbitone and phenytoin caused a significant increase in liver weight expressed as a percentage of total body weight, and a significant decrease in pentobarbitone sleeping-time compared to the vehicle controls ($P < 0{\cdot}001$). Interestingly, neither saline nor HBSS had any significant effect on liver weight compared to untreated controls ($P > 0{\cdot}1$) but both caused a slight decrease in pentobarbitone sleeping-time which was reproducible but not always statistically significant (Table II).

*Effects of phenobarbitone and phenytoin on MIS pharmacokinetics in normal BALB/c mice*

The effects of pretreatment with phenobarbitone and phenytoin (and the appropriate drug vehicles) on the plasma concentrations of MIS and Ro 05-9963 in normal BALB/c male mice are demonstrated in Figs. 1 and 2. Data showing the effects on various pertinent pharmacokinetic parameters are summarized in Tables III and IV. Results are presented for duplicate experiments, to demonstrate that although the reproducibility was generally good, some quantitative differences between experiments were obtained.

*Peak MIS concentration.*—Tables III and IV show that, in general, peak plasma MIS concentrations were not significantly affected by pretreatment with phenobarbitone, phenytoin or the drug vehicles ($P > 0{\cdot}1$). The differences in Experiment F appear to be due to a rather high peak in the vehicle controls.

*Apparent MIS half-life.*—In most experiments, the apparent $t_{\frac{1}{2}}$ for MIS in blood or plasma was somewhat reduced by pretreatment with drug vehicle, but this was not always statistically significant (Figs. 1A and 2A and Tables III and IV). Compared to the vehicle controls the apparent $t_{\frac{1}{2}}$ was further reduced by phenobarbitone and phenytoin, and this effect was always significant ($P < 0{\cdot}02$).

*Ro* 05-9963 *concentration.*—Figs. 1 and 2 show that the decrease in MIS $t_{\frac{1}{2}}$ caused by the microsomal-enzyme inducers is associated with a concomitant 1·5–2-fold increase in the blood or plasma concentrations of the O-demethylated metabolite Ro 05-9963 from $\sim \frac{1}{2}$–4 h. At later times, the metabolite concentration falls lower than the controls. In contrast, the Ro 05-9963 concentrations in the vehicle

FIG. 1.—Concentrations of MIS (A) and Ro 05-9963 (B) in plasma of control normal BALB/c mice (○) and those pretreated with phenobarbitone (△) or saline (●) after 1 g/kg MIS i.p. Error bars indicate 2 × s.e.

FIG. 2.—Concentrations of MIS (A) and Ro 05-9963 (B) in blood of control normal BALB/c mice (○) and those pretreated with phenytoin (△) or saline (●) after 1 g/kg MIS i.p. Error bars indicate 2 × s,e.

controls were identical to those in the untreated groups. Similar results were obtained in several repeat experiments.

*Area under the curve.*—Tables III and IV show that the blood or plasma $AUC_{0-8h}$ for MIS in the vehicle controls was similar to, or slightly lower than, that for the untreated groups. In mice pretreated with phenobarbitone and phenytoin the $AUC_{0-8h}$ for MIS was considerably lower than in the vehicle controls.

$AUC_{0-8h}$ values for the metabolite Ro 05-9963 were similar for untreated and vehicle controls, but rather higher in the induced mice. In general, the $AUC_{0-8h}$ for total 2-nitroimidazole (MIS + Ro 05-9963) was similar in the untreated and vehicle controls, but reduced in the induced mice.

*Effects of phenobarbitone and phenytoin on MIS pharmacokinetics in BALB/c mice bearing the EMT6 tumour*

In the next series of experiments we investigated the effects of the microsomal-enzyme inducers on BALB/c mice bearing EMT6 tumours. Concentrations of MIS and the metabolite Ro 05-9963 were determined in tumour, brain and plasma of mice pretreated with phenobarbitone, phenytoin or vehicle alone.

*Phenobarbitone.*—The effects of phenobarbitone are summarized in Figs. 3 and 4 and Table V. Similar data were obtained in a repeat experiment. Comparison of the plasma data in Table V with those in Table III shows that the pharmacokinetics are similar in normal and tumour-bearing mice, and that phenobarbitone has the same effect in both.

Comparison of tumour, brain and plasma levels in control and treated mice yielded some interesting findings which are discussed below.

Fig. 3 shows that for both saline and phenobarbitone pretreated groups, tumour MIS concentrations are similar to the corresponding brain concentrations during the period $\frac{1}{2}$–6 h after injection. The mean ($\pm$ s.e.) tumour/plasma ratios in saline and phenobarbitone pretreated mice were $0{\cdot}54 \pm 0{\cdot}09$ and $0{\cdot}50 \pm 0{\cdot}10$ respectively. Corresponding values for the brain/plasma ratios were $0{\cdot}51 \pm 0{\cdot}04$ and $0{\cdot}48 \pm 0{\cdot}09$ for saline and phenobarbitone groups respectively. However, the 15min data showed consistently that the tumour equilibrates with the plasma more slowly than does the brain.

As expected from the constant tissue/plasma ratios, the peak MIS concentra-

FIG. 3.—Concentrations of MIS in plasma (○, μg/ml), EMT6 tumour (●, μg/g) and brain (△, μg/g) of BALB/c mice pretreated with saline (A) or phenobarbitone (B). Error bars indicate typical 2 × s.e. MIS 1 g/kg i.p.

FIG. 4.—Concentrations of Ro 05-9963 in plasma (○, μg/ml), EMT6 tumour (●, μg/g) and brain (△, μg/g) of BALB/c mice pretreated with saline (A) or phenobarbitone (B). Error bars at 2 h indicate typical 2 × s.e. MIS dose 1 g/kg i.p.

tions and $AUC_{0-6h}$ values for MIS in tumour and brain were about 50% of the corresponding plasma values, whereas the apparent $t_{\frac{1}{2}}$ values for MIS in plasma and corresponding brain and tumour were not significantly different ($P > 0{\cdot}1$, Table V).

In contrast to the above findings for MIS, dissimilar results were obtained for the concentrations of the metabolite Ro 05-9963 in tumour and brain. Tumour/plasma ratios for Ro 05-9963 were similar to those for MIS; mean values ± s.e. were 0·59 ± 0·04 and 0·49 ± 0·03 for saline and phenobarbitone groups respectively. The brain/plasma ratios for the metabolite, on the other hand, were generally about half those for the parent drug; mean values (± s.e.) were 0·24 ± 0·05 and 0·27 ± 0·01 for saline and phenobarbitone groups respectively. As a consequence, the $AUC_{0-6h}$ for Ro 05-9963 in the brain was considerably lower than that in the tumour (Table V).

The effects of pretreatment with phenobarbitone on the kinetic parameters for brain and tumour (Table V) can be summarized as follows:

(a) No alteration of the tissue/plasma nitroimidazole ratios.

(b) No significant effect on peak MIS concentrations in brain and tumour ($P > 0{\cdot}1$).

(c) Significant reduction of the apparent $t_{\frac{1}{2}}$ for the elimination of MIS from brain and tumour ($P < 0{\cdot}001$).

(d) Reduction of the $AUC_{0-6h}$ for both MIS and total 2-nitroimidazole in brain and tumour.

*Phenytoin.*—Two experiments were performed on EMT6 tumour-bearing mice, and the combined data are summarized in Table VI. Comparison with Table IV shows that, like phenobarbitone, phenytoin has similar effects on normal and tumour-bearing mice.

Comparison of Tables V and VI reveals that, in general, the effects of phenytoin on the pharmacokinetic parameters for brain and tumour tissues were very similar to those caused by phenobarbitone. However, two points are worthy of note. Firstly, the tissue/plasma MIS ratios in these experiments were around 0·3, which is rather lower than in the phenobarbitone experiments (Table V). Secondly, there is an indication that phenytoin pretreatment increased the $AUC_{0-6h}$ for Ro 05-9963 in the brain and tumour tissues, an effect not seen with phenobarbitone (Table V).

*Effects of phenobarbitone on MIS pharmacokinetics in B10 mice bearing the MC6B tumour*

Two experiments were carried out to determine the effects of phenobarbitone in B10 mice bearing the MC6B tumour. The combined data are summarized in Fig. 5 and Table VII.

The results were very similar to those obtained for BALB/c mice bearing the EMT6 tumour. There are, however, 3 interesting differences.

Firstly, the apparent $t_{\frac{1}{2}}$ values for MIS elimination are rather longer, and the Ro 05-9963 metabolite concentrations correspondingly lower, than those obtained for BALB/c mice.

Secondly, phenobarbitone pretreatment caused an increase in $AUC_{0-6h}$ for the metabolite Ro 05-9963 in brain and tumour tissues. This was seen with phenytoin but not phenobarbitone in the BALB/c strain.

Thirdly, the tissue/plasma ratios with the B10 strain were higher than those in BALB/c mice. The mean ($\pm$s.e.) tumour/plasma ratios for MIS in saline and phenobarbitone pretreated mice were 0·73 $\pm$0·02 and 0·71$\pm$0·02 respectively. Corresponding values for the brain/plasma ratios for saline and phenobarbitone groups were 0·66$\pm$0·04 and 0·68$\pm$0·03 respectively. The mean tumour/plasma ratios for the metabolite Ro 05-9963 in saline and phenobarbitone groups were 0·88$\pm$0·04 and 0·72$\pm$0·06 respectively. Corresponding values for the brain/plasma ratio were 0·37$\pm$0·11 and 0·25$\pm$0·03 for saline and phenobarbitone groups respectively.

*Effects of phenobarbitone and phenytoin on urinary excretion of MIS and metabolites*

The effects of pretreatment with phenobarbitone, phenytoin and saline vehicle on the 24h urinary excretion of MIS and its metabolites are summarized in Table VIII. Of the administered 1 g/kg dose, 15–42% was recovered as MIS, Ro 05-9963 and their respective glucuronides. Ro 05-9963 glucuronide was present in lower amounts than the other nitroimidazoles. There appear to be no obvious differences in urinary excretion between the various groups.

FIG. 5.—Concentrations of MIS (A) and Ro 05-9963 (B) in blood of B10 mice (with MC6B tumours) pretreated with saline (○) or phenobarbitone (●). Error bars indicate 2 × s.e. MIS dose 1 g/kg i.p.

TABLE VIII.—*Effect of various pretreatments on urinary excretion in BALB/c mice after 1 g/kg MIS*

| Pretreatment | % Administered dose excreted in 24h urine* | | | | | | |
|---|---|---|---|---|---|---|---|
| | MIS | | | Ro 05-9963 | | | MIS + Ro 05-9963 + Glucuronides |
| | Free | Glucuronide | Total | Free | Glucuronide | Total | |
| None | 12, 8 | 6, 5 | 18, 13 | 12, 9 | 0·2, 1 | 12, 10 | 30, 23 |
| Saline | 14, 12 | 12, 6 | 26, 18 | 11, 10 | 5, 1 | 16, 11 | 42, 29 |
| Phenobarb. | 12, 11, 4 | 10, 6, 2 | 22, 17, 8 | 16, 16, 7 | 4, 1, 0·1 | 20, 17, 7 | 42, 34, 15 |
| Phenytoin | 9, 7 | 7, 4 | 16, 11 | 12, 8 | 2, 1 | 14, 9 | 30, 20 |

* Data for 2–3 independent determinations.

### *Effects of phenobarbitone and phenytoin on MIS-induced temperature loss*

Little change in body temperature was seen when BALB/c mice pretreated with saline, phenobarbitone or phenytoin were injected with 40 ml/kg HBSS (Fig. 6). Also, the temperature profiles were identical to those for mice receiving neither pretreatment nor HBSS (data not shown). In contrast, all 3 pretreated groups showed a marked decrease in temperature after 1 g/kg MIS. However, compared to the saline group, the phenobarbitone and phenytoin groups exhibited a slightly smaller decrease, and a much quicker return to normal temperature. Similar results were obtained in several repeat experiments.

FIG. 6.—Effect of various pretreatments on body temperature of normal BALB/c mice with time after injection of Hanks' solution (open symbols) or 1 g/kg MIS (closed symbols). After pretreatment with saline (○, ●) phenytoin (△, ▲) or phenobarbitone (□, ■). ▽—·—▽, ambient temperature.

### *Effect of phenobarbitone and phenytoin on MIS acute $LD_{50}$*

Two separate experiments were carried out to investigate the effect of pretreatment with phenobarbitone, phenytoin and the saline vehicle on the acute $LD_{50(7d)}$ of MIS in BALB/c males. Similar results were obtained in the two experiments, and the analysis of the combined data is summarized in Table IX. It may be seen that whereas the saline vehicle was without effect ($P > 0·1$), both phenobarbitone and phenytoin caused the $LD_{50}$ to be significantly increased ($P < 0·001$).

### DISCUSSION

In the present paper, we have shown that pretreatment of mice with phenobarbitone or phenytoin profoundly affects

TABLE IX.—*Acute $LD_{50(7d)}$ for MIS in BALB/c male mice after various pretreatments*

| Pretreatment | $LD_{50(7d)}$ (95% confidence limits) (g/kg) |
|---|---|
| None | 1·58 (1·50–1·65) |
| Saline | 1·54 (1·48–1·60)† |
| Phenobarbitone | 1·90 (1·77–2·03)* |
| Phenytoin | 1·78 (1·71–1·85)* |

10–11 different doses of MIS were used for each pretreatment group, and 5–10 mice were used at each dose.

† Not sig. diff. from untreated controls ($P > 0·1$).

the pharmacokinetics of the hypoxic cell radiosensitizer MIS. Pretreatment with these agents shortened the apparent $t_{\frac{1}{2}}$ for MIS elimination from blood or plasma by 35–60%, and this was associated with a concomitant 1·5 to 2-fold increase in the circulating concentrations of the O-demethylated metabolite Ro 05-9963. Both phenobarbitone and phenytoin are known potent inducers *in vivo* of hepatic microsomal drug-metabolizing enzymes, particularly the mixed-function oxidases which catalyse, among many other reactions, the O-demethylation of xenobiotics (Conney, 1967; Parke, 1968). We also observed that pretreatment with phenobarbitone or phenytoin caused the liver/body weight ratio to be increased, and barbiturate sleeping-time to be decreased. These are among the classical effects of agents which elevate microsomal mixed-function oxidase activities *in vivo* (Marshall & McLean, 1969; Stevenson & Turnbull, 1968; Gerber & Arnold, 1969). The preceding data therefore strongly suggest that the reduced MIS $t_{\frac{1}{2}}$ after pretreatment with phenobarbitone or phenytoin is due to the increased metabolism of MIS to Ro 05-9963 by hepatic microsomal mixed-function oxidases.

Previous studies at the same drug dose (1 g/kg) have shown that the $t_{\frac{1}{2}}$ of MIS is prolonged after bilateral kidney ligation (Brown *et al.*, 1979). Taken together with the present data, it can be seen that the MIS $t_{\frac{1}{2}}$ at this dose is dependent upon both metabolism and urinary excretion. It is interesting to note, however, that the urinary excretion of MIS, Ro 05-9963, and their respective glucuronides, was not affected by phenobarbitone or phenytoin. Urinary excretion of these compounds accounted for only 15–42% of the administered dose (1 g/kg), and this was similar to the value reported by Flockhart *et al.* (1978*a*) for normal mice given 100 mg/kg. It is apparent that changes in MIS $t_{\frac{1}{2}}$ after enzyme induction are not reflected in the urinary excretion profile. This may be due to the involvement of other metabolic pathways which may not be rate-limiting with respect to the systemic elimination of MIS.

We have seen that MIS pharmacokinetics are similar in normal and EMT6 tumour-bearing BALB/c mice. Moreover, the MIS $t_{\frac{1}{2}}$ was decreased after enzyme induction both in BALB/c mice with EMT6 tumours and B10 mice with MC6B tumours. This is of interest in view of previous reports (see Sladek *et al.*, 1978) that hepatic microsomal mixed-function oxidase activity may be reduced in animals with primary or transplanted solid tumours.

Despite the marked decrease in MIS $t_{\frac{1}{2}}$ after phenobarbitone or phenytoin, the peak blood (or plasma) MIS concentrations were reduced only slightly, if at all. In contrast, the blood (or plasma) MIS AUC was consistently decreased by 25–60%. The enhanced metabolism of MIS caused by the enzyme inducers resulted in an increased blood (or plasma) AUC for the metabolite Ro 05-9963, amounting to 10–60% in BALB/c mice and 100% in the B10 strain. Despite this increase, the blood (or plasma) AUC for total 2-nitroimidazole (MIS + Ro 05-9963) was always reduced by 20–40% after enzyme induction.

We have shown that EMT6 tumour/plasma ratios for both MIS and Ro 05-9963 were constant (within experiments) at ~0·3–0·6. Values obtained for the MC6B tumour were also constant, though higher (~0·7). For both tumours, we found that the tumour/plasma ratios were not affected when the MIS $t_{\frac{1}{2}}$ was shortened after enzyme induction. This complements the previous demonstration that this ratio was unaltered when the $t_{\frac{1}{2}}$ for MIS or injected Ro 05-9963 was *prolonged* after kidney ligation (Brown *et al.*, 1979).

The brain/plasma ratios for both MIS and Ro 05-9963 are also constant, and likewise unaffected by the decreased MIS $t_{\frac{1}{2}}$ following microsomal enzyme induction. For MIS, tumour and brain concentrations were very similar, except that the tumours equilibrated less rapidly with the plasma than did the brain. This may be due to poor tumour vascularization relative to the high cerebral blood flow. In contrast to MIS, concentrations of Ro 05-9963 were considerably lower in brain than tumour. This is presumably related to the fact that Ro 05-9963 is considerably less lipophilic than MIS (Adams *et al.*, 1976) since lipophilicity is the major factor affecting the penetration of unionized compounds of low molecular weight across the blood–brain barrier (reviewed by Bradbury & Davson, 1964). This would suggest that if brain concentration contributes to the neurotoxicity of nitroimidazoles (see below) Ro 05-9963 might be less toxic than MIS. In mice, Ro 05-9963 has a higher $LD_{50}$ than MIS, and at equal tumour concentrations they are equally good radiosensitizers (Brown *et al.*, 1979). Ro 05-9963 therefore may have potential for clinical use.

As for the peak plasma concentrations, phenobarbitone and phenytoin did not alter the peak MIS concentrations for either tumour or brain. However, apparent $t_{\frac{1}{2}}$ values for the elimination of MIS from these tissues were shortened. Moreover, the AUC for both MIS and total 2-nitroimidazole were reduced, although the AUC for Ro 05-9963 was increased in some experiments.

Having established that the pharmacokinetics of MIS are indeed altered by phenobarbitone and phenytoin the question arises as to whether these interactions alter the toxic and therapeutic effects of MIS. Taking first the radiosensitization of hypoxic cells by MIS, there is fairly good evidence from animal experiments that this property is a function of the concentration of intact nitroimidazole in the tumour at the time of radiation (McNally *et al.*, 1978). We have shown that the peak tumour MIS concentration is not altered by the enzyme inducers. Thus if radiation is given at the time of the peak concentration, the radiosensitization should not be affected. Of course, this presupposes that pretreatment with the enzyme inducers dose not have an adverse effect on the radiation response involving mechanisms unrelated to the effects on MIS disposition kinetics.

For the present discussion it is convenient to consider 3 types of toxicity displayed by MIS and related nitroheterocyclics:

(1) Cytotoxicity. Nitroheterocyclics exhibit cytotoxic properties with a marked selectivity against hypoxic cells (reviewed by Foster, 1978).
(2) Neurotoxicity. Peripheral neuropathy is the dose-limiting toxicity of MIS in man. (Dische *et al.*, 1977; Urtasun *et al.*, 1978).
(3) Lethality. Acute $LD_{50}$ assays in mice are commonly used to assess drug toxicity.

It is not clear what molecular species may be responsible for these toxic effects. Biotransformation may be involved, and Fig. 7 summarizes the probable oxidative and reductive reactions involved in the Phase 1 metabolism of MIS *in vivo*. The Phase 2 reactions, involving glucuronide conjugations, are unlikely to be of interest, since the conjugates will be unable to penetrate cell membranes and so are rapidly excreted.

It appears that oxidative metabolism has not been considered previously as contributing to MIS toxicity. O-demethylation is catalysed by microsomal mixed-function oxidases, proceeding via the intermediate methylol which breaks down spontaneously to the demethylated metabolite and formaldehyde (Fig. 7A) (Parke, 1968). Previous studies with melamines, such as hexamethylmelamine (HMM) implicated the N-methylols or formaldehyde as possible toxic species (Rutty & Connors, 1977; Rutty *et al.*, 1978). This is especially pertinent as HMM also causes neurotoxicity in man. It is also relevant that the hydroxymethyl metabolite of metronidazole is considerably more mutagenic than the parent drug (Connor *et al.*, 1977). However, the selective cytotoxicity of MIS against hypoxic cells apparently excludes oxidative metabolism from the cytotoxic mechanism. Moreover, we have shown that when the brain and plasma concentrations of the O-demethylated metabolite Ro 05-9963 are raised by enzyme induction, the $LD_{50}$ was actually increased. This suggests that oxidative metabolism is also not responsible for MIS lethality. If death is due to neurological damage, this may likewise be true for the neurotoxicity. The decreased body temperature may involve a neurological

FIG. 7.—Oxidative and reductive metabolism of MIS.

mechanism, and it is interesting that this was more short-lived after enzyme induction.

It is widely held that the selective cytotoxicity of nitroheterocyclics against hypoxic cells implicates reductive metabolism in the cytotoxic mechanism (Wardman, 1977; Willson, 1977; Foster, 1978). Reduction of the nitro group to the amine will proceed *via* the nitroradical anion, nitroso, hydroxylamine and other potentially cytotoxic intermediates (Fig. 7B) (Wardman, 1977; Willson, 1977; Whitmore *et al.*, 1978). The unstable amine has been detected in mouse tumours and human urine (Flockhart *et al.*, 1978*a*, *b*; Varghese *et al.*, 1976). Although nitroreduction is probably responsible for MIS cytotoxicity, its involvement in the neurotoxic and lethal effects is unclear. It is, however, pertinent to speculate on the possible interactions of microsomal-enzyme inducers.

Treatment with microsomal-enzyme inducers causes increased concentrations of cytochrome P-450, NADPH-cytochrome-c reductase and phosphatidyl choline, which together comprise the mixed-function oxidase enzyme complex (Conney, 1967; Lu & West, 1978). Cytochrome P-450 and NADPH-cytochrome-c reductase catalyse both oxidative and reductive reactions, including the reduction of nitroheterocyclics (Gillette, 1971, 1977). These latter reactions are also carried out by soluble enzymes, including aldehyde oxidase and xanthine oxidase (Gillette, 1971, 1977) and collectively these enzymes comprise the tissue "nitroreductase".

Phenobarbitone pretreatment *in vivo* increases nitroreduction by liver microsomes incubated under anoxic conditions *in vitro* (Conney, 1967). However, this is strongly inhibited by oxygen and may not occur in well oxygenated normal tissues *in vivo* (Gillette, 1971). Thus it is unlikely that microsomal-enzyme inducers will increase nitroreduction in normal tissues. In fact, the increased oxidative metabolism is more likely to protect against any cytotoxicity operating *via* nitroreduction.

Decreased nitroreduction may be a factor involved in the increased MIS $LD_{50}$ in mice pretreated with phenobarbitone and phenytoin. However, two other possibilities should be considered. Firstly, pretreatment with these agents may produce a physiological tolerance to MIS unrelated to metabolic factors. However, the involvement of such an effect is normally postulated only in cases where metabolic factors cannot be implicated, whereas in the present studies an increased oxidative metabolism has been clearly demonstrated. The second, and more likely, possibility is that the AUC for MIS (or total 2-nitroimidazole) may be responsible for the lethal effect: in this case the increased $LD_{50}$ would be explained by the decreased AUC. Likewise, the more rapid clearance of MIS from the brain may explain the quicker return to normal body temperature.

There is some suggestion that the dose-limiting neuropathy of MIS in man is related to the AUC (Dische *et al.*, 1977; Saunders *et al.*, 1978). If so, the toxicity might be reduced by using microsomal enzyme inducers to decrease the AUC.

It is certain that many cancer patients receiving MIS will also require other medications, including microsomal-enzyme inducers. Phenobarbitone and phenytoin, for example, are frequently administered to brain-tumour patients. The present studies provide pharmacological evidence that the radiosensitization by MIS is unlikely to be reduced by such induction and, in addition, that the toxicity might be decreased. Our preliminary studies in man suggest that the MIS $t_{\frac{1}{2}}$ and AUC are both reduced by phenytoin therapy.

I wish to thank Professor N. M. Bleehen for his advice and continued support; Dr Nancy Smith and Ms Jane Donaldson for expert technical assistance; Ms Davina Honess, Ms Jane Morgan and Ms Paula Rayner for help in some of the animal experiments; Dr J. M. Brown, Dr P. R. Twentyman and Mr L. S. Freedman for helpful discussions; and Dr C. E. Smithen of Roche Products Limited for supplies of nitroimidazoles.

### REFERENCES

ADAMS, G. E., FLOCKHART, I. R., SMITHEN, C. E., STRATFORD, I. J., WARDMAN, P. & WATTS, M. E. (1976) Electron-affinic sensitisation. VII. A correlation between structures, one-electron reduction potentials and efficiencies of nitroimidazoles as hypoxic cell radiosensitisers. *Radiat. Res.*, **67**, 9.

BRADBURY, M. W. & DAVSON, H. (1964) The blood-brain barrier. In *Absorption and Distribution of Drugs*. Ed. T. B. Binns. Edinburgh: Livingstone. p. 77.

BROWN, J. M., YU, N. Y. & WORKMAN, P. (1979) Pharmacokinetic considerations in testing hypoxic cell radiosensitizers in mouse tumours. *Br. J. Cancer*, **39**, 310.

CONNOR, C. H., STOECKEL, M., EVARD, J. & LEGATOR, M. S. (1977) The contribution of metronidazole and two metabolites to the mutagenic activity detected in urine of treated humans and mice. *Cancer Res.*, **37**, 629.

CONNEY, A. H. (1967) Pharmacological implications of microsomal enzyme induction, *Pharmacol. Rev.*, **19**, 317.

DISCHE, S., SAUNDERS, M. I., LEE, M. E., ADAMS, G. E. & FLOCKHART, I. R. (1977) Clinical testing of the radiosensitiser Ro 07-0582: Experience with multiple doses. *Br. J. Cancer*, **35**, 567.

FLOCKHART, I. R., LARGE, P., TROUP, D., MALCOM, S. L. & MARTEN, T. R. (1978*a*) Pharmacokinetic and metabolic studies of the hypoxic cell radiosensitiser misonidazole. *Xenobiotica*, **8**, 97.

FLOCKHART, I. R., MALCOM, S. L., MARTEN, T. R., PARKINS, C. S., RUANE, R. J. & TROUP, D. (1978*b*) Some aspects of the metabolism of misonidazole. *Br. J. Cancer*, **37**, Suppl. III, 264.

FOSTER, J. L. (1978) Differential cytotoxic effects of metronidazole and other nitro-heterocyclic drugs against hypoxic tumour cells. *Int. J. Radiat. Oncol. Biol. Phys.*, **4**, 153.

GERBER, N. & ARNOLD, K. (1969) Studies on the metabolism of diphenylhydantoin in mice. *J. Pharmacol. Exp. Ther.*, **167**, 77.

GILLETTE, J. R. (1971) Reductive enzymes. In *Handbook of Experimental Pharmacology*. Eds B. B. Brodie & J. R. Gillette. New York: Springer-Verlag. p. 349.

GILLETTE, J. R. (1977) Bioactivation of nitroheterocyclic compounds to chemically reactive metabolites and superoxide. In *Metronidazole*. Eds S. M. Finegold, J. A. McFadzean & F. C. J. Roe. Amsterdam: Excerpta Medica. p. 20.

GRAHAME-SMITH, D. G. (1977) (Ed.) *Drug Interactions*. London: Macmillan Press.

LU, A. Y. H. & WEST, S. B. (1978) Reconstituted mammalian mixed-function oxidases: Requirements, specificities and other properties. *Pharmacol. Ther.* (A), **2**, 337.

MARSHALL, W. J. & McLEAN, A. E. M. (1969) The effect of oral phenobarbitone on hepatic microsomal cytochrome P-450 and demethylation activity in rats fed normal and low protein diets. *Biochem. Pharmacol.*, **18**, 153.

McNALLY, N. J., DENEKAMP, J., SHELDON, P. W., FLOCKHART, I. R. & STEWART, F. A. (1978) Radiosensitisation by misonidazole (Ro 07-0582). The importance of timing and tumour concentration of sensitiser. *Radiat. Res.*, **73**, 568.

MORSELLI, P. L., COHEN, S. N. & GARATTINI, S. (1974) (Eds). *Drug Interactions*. New York: Raven Press.

PARKE, D. V. (1968) Metabolic transformations catalysed by hepatic microsomal enzymes. In *The Biochemistry of Foreign Compounds*. Oxford: Pergamon. p. 34.

PIRTTIAHO, H. I., SOTANIEMI, E. A., AHOKASJT & PITKANERI, U. (1978) Liver size and indices of drug metabolism in epileptics. *Br. J. Clin. Pharmacol.*, **6**, 273.

ROCKWELL, S. C., KALLMAN, R. F. & FAJARDO, L. F. (1972) Characteristics of a serially transplanted mouse mammary tumour and its tissue culture adapted derivative. *J. Natl Cancer Inst.*, **49**, 735.

RUTTY, C. J. & CONNORS, T. A. (1977) *In vitro* studies with hexamethylmelamine. *Biochem. Pharmacol.*, **26**, 2385.

RUTTY, C. J., CONNORS, T. A., NGUYEN-HOANG-NAM, DO-CAO-THANG & HOELLINGER, H. (1978) *In vivo* studies with hexamethylmelamine. *Eur. J. Cancer*, **14**, 713.

SAUNDERS, M. I., DISCHE, S., ANDERSON, P. & FLOCKHART, I. R. (1978) Neurotoxicity of misonidazole and its relationship to dose, half-life and concentration in the serum. *Br. J. Cancer*, **37**, Suppl. III, 286.

SIKORA, K., STERN, P. & LENNOX, E. (1977) Immunoprotection by embryonal carcinoma cells for methylcholanthrene-induced murine sarcomas. *Nature*, **269**, 813.

SLADEK, N. E., DOMEGER, B. E., MERRIMAN, R. L. & BIOPHY, G. T. (1978) Differential effects of Walker 256 carcinosarcoma cells growing subcutaneously, intramuscularly, or intraperitoneally on hepatic microsomal mixed-function oxygenase activity. *Drug Metab. Dispos.*, **6**, 412.

STEVENSON, I. H. & TURNBULL, M. R. (1968) Hepatic drug-metabolising enzyme activity and duration of hexobarbitone anaesthesia in barbitone-dependent and withdrawn rats. *Biochem. Pharmacol.*, **17**, 2297.

TWENTYMAN, P. R. & BLEEHEN, N. M. (1975) Studies of "potentially lethal damage" in EMT6 mouse tumour cells treated with bleomycin either *in vitro* or *in vivo*. *Br. J. Cancer*, **32**, 491.

URTASUN, R. C., CHAPMAN, J. D., FELDSTEIN, M. L. & 6 others (1978) Peripheral neuropathy related to misonidazole: Incidence and pathology. *Br. J. Cancer*, **37**, Suppl. III, 271.

VARGHESE, A. J., GULYAS, S. & MOHINDRA, J. K. (1976) Hypoxia-dependent reduction of 1-(2-nitro-1-imidazolyl)-3-methoxy-2-propanol by Chinese hamster ovary cells and KHT tumour cells *in vitro* and *in vivo*. *Cancer Res.*, **36**, 3701.

WARDMAN, P. (1977) The use of nitroaromatic compounds as hypoxic cell radiosensitisers. *Curr. Top. Radiat. Res.*, **11**, 347.

WARREN, R. D. & BENDER, R. A. (1977) Drug interactions with antineoplastic agents. *Cancer Treat. Rep.*, **61**, 1231.

WATSON, J. V. (1976) The cell proliferation kinetics of the EMT6/M/AC mouse tumour at four volumes during unperturbed growth. *Cell Tissue Kinet.*, **9**, 147.

WHITE, R. A. S., WORKMAN, P., FREEDMAN, L. S., OWEN, L. N. & BLEEHEN, N. M. (1979) The pharmacokinetics of misonidazole in the dog. *Eur. J. Cancer* (in press).

WHITMORE, G. F., GULYAS, S. & VARGHESE, A. J. (1978) Sensitising and toxicity properties of misonidazole and its derivatives. *Br. J. Cancer*, **37**, Suppl. III, 115.

WILLSON, R. L. (1977) Metronidazole (Flagyl) in cancer radiotherapy: A historical introduction. In *Metronidazole*. Eds S. M. Finegold, J. A. McFadzean & F. C. J. Roe. Amsterdam: Excerpta Medica. p. 20.

WORKMAN, P., WILTSHIRE, C. R., PLOWMAN, P. N. & BLEEHEN, N. M. (1978*a*) Monitoring of salivary misonidazole in man: A possible alternative to plasma monitoring. *Br. J. Cancer*, **38**, 709.

WORKMAN, P., LITTLE, C. J., MARTEN, T. R. & 4 others (1978*b*) Estimation of the hypoxic cell sensitiser misonidazole and its O-demethylated metabolite in biological materials by reversed-phase high-performance liquid chromatography. *J. Chromatogr.*, **147**, 507.